# Free Banach lattices under convexity conditions

**DOI:** 10.1007/s13398-021-01155-8

**Published:** 2021-10-11

**Authors:** Héctor Jardón-Sánchez, Niels Jakob Laustsen, Mitchell A. Taylor, Pedro Tradacete, Vladimir G. Troitsky

**Affiliations:** 1 Department of Mathematics and Statistics, Fylde College, Lancaster University, Lancaster, LA1 4YF UK; 2 Department of Mathematics, University of California, Berkeley, CA 94720 USA; 3 Instituto de Ciencias Matemáticas (CSIC-UAM-UC3M-UCM) Consejo Superior de Investigaciones Científicas, C/ Nicolás Cabrera, 13–15, Campus de Cantoblanco UAM, 28049 Madrid Spain; 4 Department of Mathematical and Statistical Sciences, University of Alberta, Edmonton, Alberta T6G 2G1 Canada

**Keywords:** Free Banach lattice, *p*-convex Banach lattice, AM-space, *p*-summing map, Primary 46B42, 46A40, 06B25, Secondary 47B60

## Abstract

We prove the existence of free objects in certain subcategories of Banach lattices, including *p*-convex Banach lattices, Banach lattices with upper *p*-estimates, and AM-spaces. From this we immediately deduce that projectively universal objects exist in each of these subcategories, extending results of Leung, Li, Oikhberg and Tursi (*Israel J. Math.* 2019). In the *p*-convex and AM-space cases, we are able to explicitly identify the norms of the free Banach lattices, and we conclude by investigating the structure of these norms in connection with nonlinear *p*-summing maps.

## Introduction

Our objective is to construct free Banach lattices having certain additional desirable properties, so let us begin by recalling the fundamental definition: The ***free Banach lattice over a Banach space*** *E* is a Banach lattice ${{\,\mathrm{\mathrm{FBL}}\,}}[E]$ together with a linear isometry $\phi _E:E\rightarrow {{\,\mathrm{\mathrm{FBL}}\,}}[E]$ such that, for every Banach lattice *X* and every bounded linear operator $T:E\rightarrow X$, there is a unique linear lattice homomorphism ${\widehat{T}}:{{\,\mathrm{\mathrm{FBL}}\,}}[E]\rightarrow X$ making the following diagram commute: 
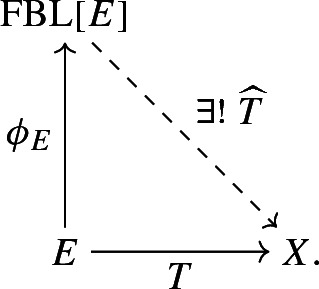


De Pagter and Wickstead [17] initiated the focused study of free Banach lattices by introducing the free Banach lattice generated by a nonempty set *A*; in the above language, it corresponds to ${{\,\mathrm{\mathrm{FBL}}\,}}\bigl [\ell _1(A)\bigr ]$. The construction of ${{\,\mathrm{\mathrm{FBL}}\,}}[E]$ for an arbitrary Banach space *E* was carried out in [7], with further research conducted in [6, 8, 18]. By now it is firmly established that free Banach lattices provide a fundamental tool for understanding the interplay between Banach-space and Banach-lattice properties. In particular, spaces of the form ${{\,\mathrm{\mathrm{FBL}}\,}}[\ell _2(A)]$ for an uncountable set *A* are used in [7, Section 5] to resolve an open problem of Diestel.

However, being universal, free Banach lattices usually lack classical properties such as reflexivity and *p*-convexity. To counteract this, we will restrict the target spaces in the above diagram to only those Banach lattices *X* which satisfy some fixed property *P*, and then look to replace ${{\,\mathrm{\mathrm{FBL}}\,}}[E]$ with a Banach lattice satisfying *P*. More specifically, we shall prove the following result.

### Theorem 1.1

Let *E* be a Banach space and $1\leqslant p\leqslant \infty$. There exists a pair $\bigl ({{\,\mathrm{\mathrm{FBL}}\,}}^{(p)}[E], \phi _E\bigr ),$ where ${{\,\mathrm{\mathrm{FBL}}\,}}^{(p)}[E]$ is a *p*-convex Banach lattice with *p*-convexity constant 1 and $\phi _E :E\rightarrow {{\,\mathrm{\mathrm{FBL}}\,}}^{(p)}[E]$ is a linear isometry, with the following universal property: For every *p*-convex Banach lattice *X* and every bounded linear operator $T:E\rightarrow X,$ there exists a unique linear lattice homomorphism ${\widehat{T}}:{{\,\mathrm{\mathrm{FBL}}\,}}^{(p)}[E]\rightarrow X$ such that ${\widehat{T}}\circ \phi _E=T$. Moreover, $\bigl \Vert {\widehat{T}}\bigr \Vert \leqslant M^{(p)}(X)\,\Vert T\Vert ,$ where $M^{(p)}(X)$ denotes the *p*-convexity constant of *X*,  and the pair $\bigl ({{\,\mathrm{\mathrm{FBL}}\,}}^{(p)}[E],\phi _E\bigr )$ is essentially unique.

In later sections we will give an explicit description of the Banach lattices whose existence is asserted in Theorem 1.1 and show analogous results when *p*-convexity is replaced with upper *p*-estimates or being a (unital) AM-space. Of course, one cannot expect a version of Theorem 1.1 for reflexivity or *p*-concavity without any restrictions on *E*, as not all Banach spaces embed into such Banach lattices.

An outline of the paper is as follows: Sect. 2 contains some preliminary material, primarily concerning function calculus, that we require in Sect. 3 when showing that ${{\,\mathrm{\mathrm{FBL}}\,}}^{(p)}[E]$ exists. In fact, the main result of Sect. 3 is somewhat more general than Theorem 1.1, as it is stated in terms of a new notion which we call “${\mathcal {D}}$-convexity” and which encompasses both *p*-convexity and upper *p*-estimates. The approach taken in Sect. 3 is similar to that of the recent paper [18], in which ${{\,\mathrm{\mathrm{FBL}}\,}}[E]$ is constructed as the completion of the free vector lattice ${\text {FVL}}[E]$ (see [9, 10]) under a certain “maximal” lattice norm. However, some additional work is needed to make sense of function calculus.

An advantage of this abstract approach is that it allows us to construct free objects in various other subcategories of Banach lattices, while a significant drawback is that it does not provide any concrete description of these spaces, notably leaving it open whether ${{\,\mathrm{\mathrm{FBL}}\,}}^{(p)}[E]$ can be realized as a vector lattice of functions. Despite this, the universal properties of these spaces are powerful enough to establish several results, which we do in Sect. 4. Section 5 is devoted to free AM-spaces and free *C*(*K*)-spaces.

Then, in Sect. 6 we return to the beginnings by giving an alternative, explicit description of ${{\,\mathrm{\mathrm{FBL}}\,}}^{(p)}[E]$ as a sublattice of the vector lattice of real-valued functions defined on the dual Banach space $E^*$ of *E*, thus in particular resolving the above problem. This section can be read independently of the previous ones.

In order to motivate the candidate norm, let us recall the construction of the space ${{\,\mathrm{\mathrm{FBL}}\,}}[E]$ and its norm from [7, Section 2]: For any function $f:E^*\rightarrow \mathbb R$, define

$$\begin{aligned} \Vert f\Vert _{{{\,\mathrm{\mathrm{FBL}}\,}}[E]}= \sup \left\{ \sum _{k=1}^n \bigl |f(x_k^*)\bigr |\,:\,n\in {\mathbb {N}}, \, x_1^*,\dots ,x_n^*\in E^*, \, \sup _{x\in B_E}\sum _{k=1}^n \bigl |x_k^*(x)\bigr | \leqslant 1\right\} . \end{aligned}$$

The set $H_1[E]$ of positively homogeneous functions $f:E^*\rightarrow {\mathbb {R}}$ with $\Vert f\Vert _{{{\,\mathrm{\mathrm{FBL}}\,}}[E]}<\infty$ turns out to be a Banach lattice with respect to the pointwise operations and this norm, and ${{\,\mathrm{\mathrm{FBL}}\,}}[E]$ is defined as the closure in $H_1[E]$ of the sublattice generated by the set $\{\delta _x \,:\,x\in E\}$, where $\delta _x:E^*\rightarrow {\mathbb {R}}$ is the evaluation map given by $\delta _x(x^*)=x^*(x)$, together with the linear isometry $\phi _E:E\rightarrow {{\,\mathrm{\mathrm{FBL}}\,}}[E]$ defined by $\phi _E(x)=\delta _x$.

We shall show that analogously, for $1<p<\infty$, ${{\,\mathrm{\mathrm{FBL}}\,}}^{(p)}[E]$ can equivalently be defined as the closure of the sublattice generated by $\{\delta _x \,:\,x\in E\}$ in the Banach lattice of positively homogeneous functions $f:E^*\rightarrow {\mathbb {R}}$ for which the quantity

1.1 $$\begin{aligned} \Vert f\Vert _p = \sup \left\{ \Bigl (\sum _{k=1}^n\bigl |f(x_k^*)\bigr |^p\Bigr )^{\frac{1}{p}} \,:\,n\in {\mathbb {N}},\, x_1^*,\dots ,x_n^*\in E^*,\, \sup _{x\in B_E}\sum _{k=1}^n\bigl |x_k^*(x)\bigr |^p\leqslant 1\right\} \nonumber \\ \end{aligned}$$

is finite. This implies in particular that there is a continuous linear injection of ${{\,\mathrm{\mathrm{FBL}}\,}}^{(p)}[E]$ into the space $C(B_{E^*})$ of continuous functions on the closed unit ball $B_{E^*}$ of $E^*$, equipped with the relative weak$^*$ topology. Although the form (1.1) of $\Vert \cdot \Vert _p$ is clearly motivated by [7], the proof that it is indeed the free *p*-convex norm requires quite different techniques. Having this explicit expression will be particularly useful in certain computations.

For the reader who is familiar with the theory of *p*-summing operators, the above expression for the free *p*-convex norm has another interpretation: A function $f:E^*\rightarrow {\mathbb {R}}$ for which $\Vert f\Vert _p<\infty$ maps weakly *p*-summable sequences in $E^*$ to (strongly) *p*-summable sequences in ${\mathbb {R}}$. In Sect. 7 we devote our attention to the spaces of positively homogeneous functions from $E^*$ to ${\mathbb {R}}$ with finite (*p*, *q*)-summing norm, and explore classical arguments such as the Dvorezky–Rogers Theorem and Pietsch’s Domination Theorem in this nonlinear setting.

## Preliminaries

Our notation and terminology are mostly standard and will be introduced as and when needed. A few general conventions are as follows. All vector spaces, including vector lattices, Banach spaces and Banach lattices, are real. The terms “operator” and “lattice homomorphism” will be synonymous with “bounded linear operator” and “linear lattice homomorphism”, respectively. A “sublattice” of a vector lattice will mean a linear subspace which is closed under finite suprema and infima. We shall repeatedly use the elementary fact that the sublattice generated by a subset *W* of a vector lattice is given by

2.1 $$\begin{aligned} \left\{ \bigvee _{j=1}^n x_j - \bigvee _{j=1}^n y_j \,:\,n\in {\mathbb {N}},\, x_1,\dots ,x_n,y_1,\dots ,y_n\in {\text {span}}W\right\} ; \end{aligned}$$

see, e.g., [3, p. 204, Exercise 8(b)].

For a positive element *e* of a vector lattice *X*, $I_e$ denotes the order ideal of *X* generated by *e*, that is,

$$\begin{aligned} I_e = \bigl \{ x\in X \,:\,|x|\leqslant \lambda e\ \text {for some}\ \lambda \in [0,\infty )\bigr \}. \end{aligned}$$

We can endow $I_e$ with the lattice seminorm defined by

2.2 $$\begin{aligned} \Vert x\Vert _e = \inf \bigl \{ \lambda \in [0,\infty ) \,:\,|x|\leqslant \lambda e\bigr \} \end{aligned}$$

for every $x\in I_e$. This seminorm is a norm if *X* is Archimedean.

### Function calculus

The definitions of *p*-convexity and upper *p*-estimates can be stated by means of function calculus, which is a standard tool in Banach lattices (see, e.g., [15, 1.d]). However, since our constructions will require us to work in more general vector lattices, we bring some basic facts to the reader’s attention. We essentially need only what is contained in [13]. For $m\in {\mathbb {N}}$, ${\mathcal {H}}_m$ denotes the vector lattice of continuous, positively homogeneous, real-valued functions on ${\mathbb {R}}^m$. Clearly it contains the $k^{\text {th}}$ coordinate projection $\pi _k:(t_1,\dots ,t_m)\mapsto t_k$ for each $k\in \{1,\dots ,m\}$. We say that a vector lattice *X* ***admits a positively homogeneous continuous function calculus*** if, for every $m\in {\mathbb {N}}$ and every *m*-tuple ${\varvec{x}}=(x_1,\dots ,x_m)\in X^m$, there is a lattice homomorphism $\Phi _{{\varvec{x}}}:{\mathcal {H}}_m\rightarrow X$ such that

2.3 $$\begin{aligned} \Phi _{{\varvec{x}}}(\pi _k) = x_k \end{aligned}$$

for each $k\in \{1,\ldots ,m\}$. In this case, we refer to the map ${\varvec{x}}\mapsto \Phi _{{\varvec{x}}}$ (or simply $\Phi _{{\varvec{x}}}$) as a ***positively homogeneous continuous function calculus*** for *X*. In line with common practice, for $h\in {\mathcal {H}}_m$, we usually use the shorter and more suggestive notation $h(x_1,\dots ,x_m)$ instead of $\Phi _{{\varvec{x}}}(h)$. It is well known that every uniformly complete vector lattice (in particular, every Banach lattice) admits a positively homogeneous continuous function calculus. The following more precise characterization was given in [13, Theorem 1.3]: An Archimedean vector lattice *X* admits a positively homogeneous continuous function calculus if and only if it is ***finitely uniformly complete*** in the sense that, for every $m\in {\mathbb {N}}$ and $x_1,\ldots ,x_m\in X$, there is a positive element $e\in X$ such that $e\geqslant \bigvee _{j=1}^m|x_j|$ and the norm $\Vert \,\cdot \,\Vert _e$ given by (2.2) is complete on the closed sublattice of $\bigl (I_e,\Vert \,\cdot \,\Vert _e\bigr )$ generated by $x_1,\ldots ,x_m$. We shall freely use this result in the following without any further reference. We can define a norm $\Vert \,\cdot \,\Vert _{{\mathcal {H}}_m}$ on ${\mathcal {H}}_m$ by identifying it with $C(S_{\ell _\infty ^m})$ via the restriction map $h\mapsto h|_{S_{\ell _\infty ^m}}$, where $S_{\ell _\infty ^m}$ denotes the unit sphere of $\ell _\infty ^m$. The sublattice of ${\mathcal {H}}_m$ generated by $\{\pi _k \,:\,1\leqslant k\leqslant m\}$ is dense with respect to this norm. We shall now establish some basic facts that we require in later sections. They may be known, but as we have been unable to find any precise references in the literature, we include their proofs. We begin with a result which will imply that when an Archimedean vector lattice admits a positively homogeneous continuous function calculus, it is unique in a very strong sense.

#### Lemma 2.1

Let $T:X\rightarrow Y$ be a lattice homomorphism between two Archimedean vector lattices *X* and *Y*,  let $x_1,\ldots ,x_m\in X$ for some $m\in {\mathbb {N}},$ and define $e = \bigvee _{j=1}^m|x_j|$ in $X_+$. Then:

(i) *T* maps the ideal $I_e$ of *X* into the ideal $I_{Te}$ of *Y*,  and $\Vert Tx\Vert _{Te}\leqslant \Vert x\Vert _e$ for every $x\in I_e$.
(ii) $\Vert Tx\Vert _{Te} = \Vert x\Vert _e$ for each $x\in I_e$ if and only if the restriction of *T* to $I_e$ is injective.

Suppose that $\Phi _{{\varvec{x}}}:{\mathcal {H}}_m\rightarrow X$ is a lattice homomorphism which satisfies (2.3). Then:

(iii) The image of $\Phi _{{\varvec{x}}}$ is contained in $I_e$, and $\Phi _{{\varvec{x}}}$ is bounded with norm at most 1 when considered an operator into $\bigl (I_e,\Vert \,\cdot \,\Vert _e\bigr )$.
(iv) The composite map $T\circ \Phi _{{\varvec{x}}}$ is the unique lattice homomorphism from ${\mathcal {H}}_m$ into *Y* which maps $\pi _k$ to $Tx_k$ for each $k=1,\ldots ,m$.

#### Proof

(i) For every $x\in I_e$, we can find $\lambda \in [0,\infty )$ such that $|x|\leqslant \lambda e$. Since *T* is a lattice homomorphism, we have $|Tx|\leqslant \lambda Te$, so $Tx\in I_{Te}$ with $\Vert Tx\Vert _{Te}\leqslant \lambda$. Now the conclusion follows by taking the infimum over all $\lambda$ with $|x|\leqslant \lambda e$.
(ii) The forward implication is clear because $\Vert \,\cdot \,\Vert _e$ is a norm on $I_e$.
  Conversely, suppose that the restriction $T|_{I_e}$ is injective, and consider $x\in I_e$ and $\lambda \in [0,\infty )$ with $|Tx|\leqslant \lambda Te$. Then $T\bigl (\lambda e - |x|\bigr ) = \lambda Te -|Tx|\geqslant 0$, so that
  $$\begin{aligned} 0 = 0\wedge T\bigl (\lambda e - |x|\bigr ) = T\Bigl (0\wedge \bigl (\lambda e - |x|\bigr )\Bigr ). \end{aligned}$$
  This implies that $0\wedge \bigl (\lambda e-|x|\bigr ) =0$ by the injectivity of $T|_{I_e}$, that is, $\lambda e\geqslant |x|$. Hence $\lambda \geqslant \Vert x\Vert _e$, and taking the infimum over all $\lambda$ with $|Tx|\leqslant \lambda Te$, we conclude that $\Vert Tx\Vert _{Te}\geqslant \Vert x\Vert _e$. The opposite inequality was shown in (i).
(iii) This is a special case of (i), applied with $T=\Phi _{{\varvec{x}}}$ and $x_k =\pi _k|_{S_{\ell _\infty ^m}}$ for each $k=1,\ldots ,m$. To see this, recall our identification of ${\mathcal {H}}_m$ with $C(S_{\ell _\infty ^m})$, and observe that $\bigvee _{k=1}^m\bigl |\pi _k|_{S_{\ell _\infty ^m}}\bigr |= \mathbb {1}$ is a strong unit in $C(S_{\ell _\infty ^m})$, with the corresponding lattice norm (2.2) being equal to the uniform norm $\Vert \,\cdot \,\Vert _\infty$.
(iv) Only the uniqueness statement is not clear. To prove it, let $S:{\mathcal {H}}_m\rightarrow Y$ be any lattice homomorphism with $S(\pi _k)=Tx_k$ for each $k=1,\ldots ,m$. By (i) and (iii), we may regard $T:I_e\rightarrow I_{Te}$, $\Phi _{{\varvec{x}}}:{\mathcal {H}}_m\rightarrow I_e$, and $S:{\mathcal {H}}_m\rightarrow I_{Te}$ as bounded lattice homomorphisms with respect to the specified domains and codomains, where $I_e$ and $I_{Te}$ are given the norms $\Vert \,\cdot \,\Vert _e$ and $\Vert \,\cdot \,\Vert _{Te}$, respectively. Then $T\circ \Phi _{{\varvec{x}}}:{\mathcal {H}}_m\rightarrow I_{Te}$ is also bounded, and therefore it is equal to *S* because $(T\circ \Phi _{{\varvec{x}}})(\pi _k) = S(\pi _k)$ for each $k=1,\ldots ,m$ and $\{\pi _k \,:\,1\leqslant k\leqslant m\}$ generates a dense sublattice of ${\mathcal {H}}_m$. $\square$

#### Corollary 2.2

Let *X* be a finitely uniformly complete Archimedean vector lattice, and let *Y* be a sublattice of *X*. Then *Y* is finitely uniformly complete if and only if $\Phi _{{\varvec{y}}}({\mathcal {H}}_m)\subseteq Y$ for every $m\in {\mathbb {N}}$ and ${\varvec{y}}\in Y^m$, where $\Phi _{{\varvec{y}}}$ denotes the positively homogeneous continuous function calculus for *X*.

#### Proof

The implication $\Leftarrow$ is clear, while the converse follows from the uniqueness statement in Lemma 2.1(iv), applied in the case where $T:Y\rightarrow X$ is the inclusion map. $\square$

#### Corollary 2.3

Let $T:X\rightarrow Y$ be a lattice homomorphism between two finitely uniformly complete Archimedean vector lattices *X* and *Y*. Then $T\bigl (h(x_1,\dots ,x_m)\bigr )=h(Tx_1,\dots ,Tx_m)$ for every $m\in {\mathbb {N}},$ $h\in {\mathcal {H}}_m,$ and $x_1,\ldots ,x_m\in X$.

#### Proof

This is immediate from Lemma 2.1(iv). $\square$

Our next result involves the following standard notion: A sequence $(x_k)$ in an Archimedean vector lattice *X* ***converges uniformly*** to $x\in X$ if *X* contains a positive element *e* such that, for every $\varepsilon \in (0,\infty )$, there is $k_0\in {\mathbb {N}}$ with $|x_k-x|\leqslant \varepsilon e$ whenever $k\geqslant k_0$. If explicit reference to the element *e* is required, we call it a ***regulator*** and say that it ***witnesses*** the convergence.

#### Lemma 2.4

Let *X* be a finitely uniformly complete Archimedean vector lattice, let $m\in {\mathbb {N}},$ and suppose that $(x_k^1),\ldots , (x_k^m)$ are sequences in *X* which converge uniformly to $x^1,\ldots ,x^m,$ respectively. Then the sequence $\bigl (h(x^1_k,\ldots ,x^m_k)\bigr )$ converges uniformly to $h(x^1,\ldots ,x^m)$ for every $h\in {\mathcal {H}}_m$.

#### Proof

For each $j=1,\ldots ,m$, let $e^j\in X_+$ be a regulator which witnesses that $(x^j_k)$ converges uniformly to $x^j$. We shall show that

2.4 $$\begin{aligned} f = \bigvee _{j=1}^m|x^j|+ \sum _{j=1}^me^j+ \bigvee _{j=1}^m(|x^j| + e^j)\in X_+ \end{aligned}$$

is a regulator witnessing that $\bigl (h(x^1_k,\ldots ,x^m_k)\bigr )$ converges uniformly to $h(x^1,\ldots ,x^m)$ for any $h\in {\mathcal {H}}_m$. To this end, let $\varepsilon >0$. Since the sublattice generated by $\{\pi _1,\ldots , \pi _m\}$ is dense in ${\mathcal {H}}_m$, it contains a function $\ell$ such that $\Vert h-\ell \Vert _{{\mathcal {H}}_m}\leqslant \varepsilon$, that is,

2.5 $$\begin{aligned} \Bigl |h(t_1,\ldots ,t_m)-\ell (t_1,\ldots ,t_m)\Bigr |\leqslant \varepsilon \bigvee _{j=1}^m|t_j| \end{aligned}$$

for every $t_1,\ldots ,t_m\in {\mathbb {R}}$. By (2.1), we can express $\ell$ as

$$\begin{aligned} \ell =\bigvee _{i=1}^n\left( \sum _{j=1}^m\alpha _{ij}\pi _j\right) -\bigvee _{i=1}^n\left( \sum _{j=1}^m\beta _{ij}\pi _j\right) \end{aligned}$$

for some $n\in {\mathbb {N}}$ and coefficients $\alpha _{ij},\beta _{ij}\in {\mathbb {R}}$. It follows that for every $k\in {\mathbb {N}}$,

2.6 $$\begin{aligned} \Bigl |\ell&(x^1,\dots ,x^m)-\ell (x_k^1,\dots ,x_k^m)\Bigr | \nonumber \\&\leqslant \biggl |\bigvee _{i=1}^n\Bigl (\sum _{j=1}^m\alpha _{ij}x^j\Bigr ) - \bigvee _{i=1}^n\Bigl (\sum _{j=1}^m\alpha _{ij}x_k^j\Bigr )\biggr | + \biggl |\bigvee _{i=1}^n\Bigl (\sum _{j=1}^m\beta _{ij}x^j\Bigr ) - \bigvee _{i=1}^n\Bigl (\sum _{j=1}^m\beta _{ij}x_k^j\Bigr )\biggr |\nonumber \\&\leqslant \bigvee _{i=1}^n\sum _{j=1}^m|\alpha _{ij}|\ |x^j - x_k^j| +\bigvee _{i=1}^n\sum _{j=1}^m|\beta _{ij}|\ |x^j-x_k^j| \leqslant C\sum _{j=1}^m|x^j - x_k^j|, \end{aligned}$$

where $C=\bigvee _{i,j}|\alpha _{ij}|+\bigvee _{i,j}|\beta _{ij}|$. Choose $k_0\in {\mathbb {N}}$ such that $|x^j - x_k^j|\leqslant \frac{\varepsilon }{\varepsilon \,\vee \,C}e^j$ for every $k\geqslant k_0$ and $j=1,\ldots ,m$. Then, combining the estimate

$$\begin{aligned}&\Bigl |h(x^1,\ldots ,x^m)-h(x_k^1,\ldots ,x_k^m)\Bigr | \leqslant \Bigl |h(x^1,\ldots ,x^m)-\ell (x^1,\ldots ,x^m)\Bigr |\\&\quad + \Bigl |\ell (x^1,\ldots ,x^m)-\ell (x_k^1,\ldots ,x_k^m)\Bigr | + \Bigl |\ell (x_k^1,\ldots ,x_k^m)- h(x_k^1,\ldots ,x_k^m)\Bigr | \end{aligned}$$

with (2.5)–(2.6), we obtain

$$\begin{aligned} \Bigl |h(x^1,\ldots ,x^m)-h(x_k^1,\ldots ,x_k^m)\Bigr | \leqslant \varepsilon \bigvee _{j=1}^m|x^j|+ \varepsilon \sum _{j=1}^me^j+ \varepsilon \bigvee _{j=1}^m|x^j_k| \leqslant \varepsilon f \end{aligned}$$

for every $k\geqslant k_0$, where *f* is defined by (2.4). This completes the proof. $\square$

#### Corollary 2.5

Let *X* be a Banach lattice, and let $h\in {\mathcal {H}}_m$ for some $m\in {\mathbb {N}}$. Then the map from $X^m$ to *X* defined via $(x_1,\ldots ,x_m)\mapsto h(x_1,\ldots ,x_m)$ is continuous with respect to the norm $\bigl \Vert (x_1,\ldots ,x_m)\bigr \Vert =\bigvee _{j=1}^m\Vert x_j\Vert$ on $X^m$.

#### Proof

Assume the contrary. Then there are sequences $(x_k^1),\ldots , (x_k^m)$ in *X* which norm converge to $x^1,\ldots ,x^m,$ respectively, but the sequence $\bigl (h(x^1_k,\ldots ,x^m_k)\bigr )$ is not norm convergent to $h(x^1,\ldots ,x^m)$. Passing to a subsequence, we may suppose that there is $\varepsilon >0$ such that

2.7 $$\begin{aligned} \Bigl \Vert h(x^1_k,\ldots ,x^m_k) - h(x^1,\ldots ,x^m)\Bigr \Vert \geqslant \varepsilon \end{aligned}$$

for every $k\in {\mathbb {N}}$. Since every norm convergent sequence has a uniformly convergent subsequence, we may further suppose that $(x_k^1),\ldots , (x_k^m)$ converge uniformly to $x^1,\ldots ,x^m,$ respectively. Then Lemma 2.4 implies that $\bigl (h(x^1_k,\ldots ,x^m_k)\bigr )$ converges uniformly to $h(x^1,\ldots ,x^m)$, which contradicts (2.7). $\square$

## Construction of free ${\mathcal {D}}$-convex Banach lattices

A Banach lattice *X* is *p*-***convex*** for some $p\in [1,\infty ]$ if there exists a constant $M\geqslant 1$ such that for every $m\in \mathbb N$ and $x_1,\dots ,x_m\in X$, we have

3.1 $$\begin{aligned} \biggl \Vert \Bigl (\sum _{k=1}^m|x_k|^p\Bigr )^{\frac{1}{p}}\biggr \Vert \leqslant M\Bigl (\sum _{k=1}^m\Vert x_k\Vert ^p\Bigr )^{\frac{1}{p}}. \end{aligned}$$

Clearly it suffices to verify this inequality in the case where $x_1,\dots ,x_m$ are positive. The least constant *M* for which (3.1) is valid is denoted $M^{(p)}(X)$ and is called the *p*-***convexity constant*** of *X*. If (3.1) is assumed to hold only for pairwise disjoint elements $x_1,\dots ,x_m\in X$, then *X* is said to satisfy an ***upper*** *p*-***estimate***. The reader is referred to [15] for further information on such Banach lattices.

In this section we present a general construction which yields the existence of both free *p*-convex Banach lattices and free Banach lattices with upper *p*-estimates. For this, we need a definition encompassing both concepts.

Let $m\in {\mathbb {N}}$, and recall that ${\mathcal {H}}_m$ denotes the set of all continuous, positively homogeneous functions from ${\mathbb {R}}^m$ to ${\mathbb {R}}$. We say that a function $h\in {\mathcal {H}}_m$ is ***monotone on*** ${\mathbb {R}}^m_+$ if $h(t_1,\dots ,t_m)\leqslant h(s_1,\dots ,s_m)$ whenever $t_1,\dots ,t_m,s_1,\dots ,s_m\in {\mathbb {R}}$ with $0\leqslant t_k\leqslant s_k$ for each $k=1,\ldots ,m$. This, of course, implies that $h(t_1,\dots ,t_m)\geqslant 0$ whenever $t_1,\dots ,t_m\geqslant 0$. We denote by ${\mathcal {H}}_m^{>0}$ the set of all continuous, positively homogeneous functions which are monotone on ${\mathbb {R}}^m_+$.

By a ***convexity condition***, we understand a triple ${\mathcal {D}}=({\mathcal {G}}, M,\vartheta )$, where ${\mathcal {G}}$ is a nonempty subset of $\bigcup _{m=1}^\infty {\mathcal {H}}_m^{>0}$ and $M:{\mathcal {G}}\rightarrow [1,\infty )$ and $\vartheta :{\mathcal {G}}\rightarrow \{0,1\}$ are any functions. Given a convexity condition ${\mathcal {D}}=({\mathcal {G}}, M,\vartheta )$, we set ${\mathcal {G}}_m = {\mathcal {G}}\cap {\mathcal {H}}_m^{>0}$ for $m\in \mathbb N$ and say that a lattice seminorm $\nu$ on a finitely uniformly complete Archimedean vector lattice *X* is ${\mathcal {D}}$-***convex*** if, for every $m\in {\mathbb {N}}$ and $g\in {\mathcal {G}}_m$, the inequality

3.2 $$\begin{aligned} \nu \bigl (g(x_1,\dots ,x_m)\bigr )\leqslant M(g)\cdot g\bigl (\nu (x_1),\dots , \nu (x_m)\bigr ) \end{aligned}$$

holds for all pairwise disjoint elements $x_1,\dots , x_m\in X_+$ if $\vartheta (g)=0$, and for all elements $x_1,\dots , x_m\in X_+$ if $\vartheta (g)=1$. A Banach lattice is ${\mathcal {D}}$-***convex*** if its norm is ${\mathcal {D}}$-convex.

Note that every closed sublattice of a ${\mathcal {D}}$-convex Banach lattice is ${\mathcal {D}}$-convex by uniqueness of the function calculus.

### Remark 3.1

It should be clear that *p*-convexity and upper *p*-estimates can be recovered easily from ${\mathcal {D}}$-convexity. Indeed, for $p\in [1,\infty ]$, let ${\mathcal {G}}^p=\bigl \{\Vert \,\cdot \,\Vert _{\ell _p^m} \,:\,m\in {\mathbb {N}}\bigr \}$ be the collection of all $\ell _p^m$-norms, and let $M:{\mathcal {G}}^p\rightarrow [1,\infty )$ be a constant function. Then, choosing $\vartheta$ to be the constant function 0, we obtain an upper *p*-estimate, while choosing $\vartheta$ to be the constant function 1 gives *p*-convexity (with constant $M^{(p)}\leqslant M$).

### Lemma 3.2

Let ${\mathcal {D}}$ be a convexity condition. The completion of a finitely uniformly complete Archimedean vector lattice with respect to a ${\mathcal {D}}$-convex lattice norm is a ${\mathcal {D}}$-convex Banach lattice.

### Proof

Suppose that ${\mathcal {D}}=({\mathcal {G}},M,\vartheta )$, and let *X* be a finitely uniformly complete Archimedean vector lattice endowed with a ${\mathcal {D}}$-convex lattice norm $\Vert \,\cdot \,\Vert$. Its completion ${\widetilde{X}}$ is a Banach lattice, so we just need to verify that ${\widetilde{X}}$ is ${\mathcal {D}}$-convex. Suppose that $g\in {\mathcal {G}}_m$ for some $m\in {\mathbb {N}}$.

We begin with the case $\vartheta (g)=1$ as it is easier. Given $x^1,\ldots ,x^m\in {\widetilde{X}}_+$, choose sequences $(x_k^1),\ldots , (x_k^m)$ in $X_+$ which converge in norm to $x^1,\ldots ,x^m$, respectively. The ${\mathcal {D}}$-convexity of the norm on *X* coupled with the continuity of *g* implies that

$$\begin{aligned} \bigl \Vert g(x_k^1,\ldots ,x_k^m)\bigr \Vert \leqslant M(g)\cdot g\bigl (\Vert x_k^1\Vert ,\ldots ,\Vert x_k^m\Vert \bigr ) \xrightarrow {k\rightarrow \infty } M(g)\cdot g\bigl (\Vert x^1\Vert ,\ldots ,\Vert x^m\Vert \bigr ). \end{aligned}$$

Now (3.2) follows because the left-hand side converges to $\bigl \Vert g(x^1,\ldots ,x^m)\bigr \Vert$ by Corollary 2.5.

Suppose instead that $\vartheta (g)=0$, and let $x^1,\ldots ,x^m\in {\widetilde{X}}_+$ be pairwise disjoint. As before, choose sequences $(y_k^1),\ldots , (y_k^m)$ in $X_+$ which converge in norm to $x^1,\ldots ,x^m$, respectively. We can “disjointify” these sequences by defining

$$\begin{aligned} x_k^i=\bigwedge \bigl \{y_k^i-y_k^i\wedge y^j_k \,:\,1\leqslant j\leqslant m,\, j\ne i\bigr \} \end{aligned}$$

for every $k\in {\mathbb {N}}$ and $i=1,\ldots ,m$. Then $x_k^1,\ldots ,x_k^m$ are pairwise disjoint for every $k\in \mathbb N$, and the sequence $(x_k^i)$ converges in norm to $x^i$ for each $i=1,\ldots ,m$ because the lattice operations are continuous and $x^1,\ldots ,x^m$ are pairwise disjoint. We can now complete the proof as in the case $\vartheta (g)=1$. $\square$

### Theorem 3.3

Let *E* be a Banach space and ${\mathcal {D}}$ a convexity condition, as defined above. There exists a pair $\bigl ({{\,\mathrm{\mathrm{FBL}}\,}}^{{\mathcal {D}}}[E],\phi _E^{\mathcal {D}}\bigr ),$ where ${{\,\mathrm{\mathrm{FBL}}\,}}^{{\mathcal {D}}}[E]$ is a ${\mathcal {D}}$-convex Banach lattice and $\phi _E^{\mathcal {D}}:E\rightarrow {{\,\mathrm{\mathrm{FBL}}\,}}^{{\mathcal {D}}}[E]$ a linear isometry, with the following universal property: For every ${\mathcal {D}}$-convex Banach lattice *X* and every operator $T:E \rightarrow X,$ there exists a unique lattice homomorphism ${\widehat{T}}:{{\,\mathrm{\mathrm{FBL}}\,}}^{{\mathcal {D}}}[E] \rightarrow X$ such that ${\widehat{T}}\circ \phi _E^{\mathcal {D}}=T,$ i.e., the following diagram commutes:

Moreover, $$\Vert {\widehat{T}}\Vert =\Vert T\Vert $$.

### Proof

Throughout this proof, we work in the Archimedean vector lattice ${\mathbb {R}}^{B_{E^*}}$ of real-valued functions defined on the closed unit ball $B_{E^*}$ of the dual space $E^*$. Being a vector lattice of functions, ${\mathbb {R}}^{B_{E^*}}$ admits a positively homogeneous continuous function calculus which is defined pointwise, that is,

3.3 $$\begin{aligned} \bigl (h(f_1,\ldots ,f_m)\bigr )(x^*) = h\bigl (f_1(x^*),\ldots ,f_m(x^*)\bigr ) \end{aligned}$$

for $$m\in {\mathbb {N}}$$, $$h\in {\mathcal {H}}_m$$, $$f_1,\ldots ,f_m\in \mathbb R^{B_{E^*}}$$, and $$x^*\in B_{E^*}$$.

Regarding the point evaluations $\delta _x:x^*\mapsto x^*(x)$ for $x\in E$ as elements of ${\mathbb {R}}^{B_{E^*}}$, we can define

3.4 $$\begin{aligned} Y_0=\Bigl \{h(\delta _{x_1},\ldots ,\delta _{x_m})\,:\,m\in {\mathbb {N}},\, h\in {\mathcal {H}}_m,\, x_1,\ldots ,x_m\in E\Bigr \}\subseteq \mathbb R^{B_{E^*}}. \end{aligned}$$

The set $\bigcup _{m=1}^\infty {\mathcal {H}}_m$ is closed under compositions in the sense that it contains the function

$$\begin{aligned} (t_1^1,\ldots ,t_{m_1}^1,\ldots ,t_1^n,\ldots ,t_{m_n}^n)\mapsto h\bigl (g_1(t_1^1,\ldots ,t_{m_1}^1),\ldots ,g_n(t_1^n,\ldots ,t_{m_n}^n)\bigr ) \end{aligned}$$

whenever $n,m_1,\ldots ,m_n\in {\mathbb {N}}$, $h\in {\mathcal {H}}_n$, and $g_1\in {\mathcal {H}}_{m_1},\ldots ,g_n\in {\mathcal {H}}_{m_n}$. This implies that $Y_0$ defined above is closed under positively homogeneous continuous function calculus. It follows in particular that $Y_0$ is a sublattice of ${\mathbb {R}}^{B_{E^*}}$ because the functions $(s,t)\mapsto s\vee t$ and $(s,t)\mapsto s+\alpha t$ for $\alpha \in {\mathbb {R}}$ both belong to ${\mathcal {H}}_2$.

Let ${\mathcal {N}}_{{\mathcal {D}}}$ denote the collection of all ${\mathcal {D}}$-convex lattice seminorms $\nu :Y_0\rightarrow [0,\infty )$ which satisfy

3.5 $$\begin{aligned} \nu (\delta _{x})\leqslant \Vert x\Vert \end{aligned}$$

for every $x\in E$, and define

3.6 $$\begin{aligned} \Vert f\Vert _{{\mathcal {D}}} =\sup \bigl \{\nu (f)\,:\,\nu \in {\mathcal {N}}_{{\mathcal {D}}}\bigr \} \end{aligned}$$

for $f\in Y_0$. We claim that this quantity is finite. Indeed, (3.4) implies that $f=h(\delta _{x_1},\dots ,\delta _{x_m})$ for some $m\in {\mathbb {N}}$, $h\in {\mathcal {H}}_m$, and $x_1,\dots ,x_m\in E$. Then, applying Lemma 2.1(iii), we obtain $\Vert f\Vert _e\leqslant \Vert h\Vert _{{\mathcal {H}}_m}$, where $e=\bigvee _{i=1}^m|\delta _{x_i}|$. By the definition (2.2) of the norm $\Vert \,\cdot \,\Vert _e$, this means that

$$\begin{aligned} |f|\leqslant \Vert h\Vert _{{\mathcal {H}}_m}e \leqslant \Vert h\Vert _{{\mathcal {H}}_m}\sum _{i=1}^m|\delta _{x_i}|, \end{aligned}$$

and therefore we have

$$\begin{aligned} \nu (f)\leqslant \Vert h\Vert _{{\mathcal {H}}_m}\sum _{i=1}^m \nu \bigl (|\delta _{x_i}|\bigr ) \leqslant \Vert h\Vert _{{\mathcal {H}}_m}\sum _{i=1}^m\Vert x_i\Vert \end{aligned}$$

for every $\nu \in {\mathcal {N}}_{{\mathcal {D}}}$. Since the right-hand side of this estimate is independent of $\nu$, we conclude that $\Vert f\Vert _{{\mathcal {D}}}$ is finite, as claimed.

Consequently, being the supremum over a family of ${\mathcal {D}}$-convex lattice seminorms, $\Vert \,\cdot \,\Vert _{{\mathcal {D}}}$ is itself a ${\mathcal {D}}$-convex lattice seminorm. It is in fact a norm, as we shall show next. For every $x^*\in B_{E^*}$, we can define a lattice seminorm $\nu _{x^*}$ on $Y_0$ by

3.7 $$\begin{aligned} \nu _{x^*}(f) =\bigl |f(x^*)\bigr |. \end{aligned}$$

Clearly $\nu _{x^*}$ satisfies (3.5). Moreover, using (3.3), we find

$$\begin{aligned} \nu _{x^*}\bigl (g(f_1,\ldots ,f_m)\bigr ) =\Bigl |g\bigl (f_1(x^*),\ldots ,f_m(x^*)\bigr )\Bigr | =g\bigl (\nu _{x^*}(f_1),\ldots ,\nu _{x^*}(f_m)\bigr ) \end{aligned}$$

for every $m\in {\mathbb {N}}$, $g\in {\mathcal {H}}_m^{>0}$ and $f_1,\ldots ,f_m\in (Y_0)_+$. This implies that $\nu _{x^*}$ is ${\mathcal {D}}$-convex because the constant *M*(*g*) in (3.2) is at least 1, and therefore $\nu _{x^*}\in {\mathcal {N}}_{\mathcal {D}}$.

Suppose that $f\in Y_0$ with $\Vert f\Vert _{{\mathcal {D}}}=0$. Then $0=\nu _{x^*}(f) = \bigl |f(x^*)\bigr |$ for every $x^*\in B_{E^*}$, so $f=0$. Hence $\Vert \,\cdot \,\Vert _{{\mathcal {D}}}$ is a ${\mathcal {D}}$-convex lattice norm on $Y_0$. We can now define ${{\,\mathrm{\mathrm{FBL}}\,}}^{{\mathcal {D}}}[E]$ as the completion of $Y_0$ with respect to this norm.

Lemma 3.2 shows that ${{\,\mathrm{\mathrm{FBL}}\,}}^{{\mathcal {D}}}[E]$ is a ${\mathcal {D}}$-convex Banach lattice. Moreover, the map $\phi _E^{{\mathcal {D}}}:\ E\rightarrow {{\,\mathrm{\mathrm{FBL}}\,}}^{{\mathcal {D}}}[E]$ defined by $\phi _E^{{\mathcal {D}}}(x) =\delta _{x}$ is clearly linear. To see that it is isometric, for $x\in E$, choose $x^*\in B_{E^*}$ such that $x^*(x) = \Vert x\Vert$. Then, using (3.5), (3.6), and (3.7), we obtain

$$\begin{aligned} \Vert \delta _{x}\Vert _{{\mathcal {D}}}\leqslant \Vert x\Vert =\nu _{x^*}(\delta _{x})\leqslant \Vert \delta _{x}\Vert _{{\mathcal {D}}}, \end{aligned}$$

so that $$\bigl \Vert \phi _E^{{\mathcal {D}}}(x)\bigr \Vert _{{\mathcal {D}}}=\Vert x\Vert $$.

It remains to verify the universal property. Let $T:E\rightarrow X$ be an operator into a ${\mathcal {D}}$-convex Banach lattice *X*. We may suppose that $\Vert T\Vert =1$. Recall from the Introduction that the original construction of ${{\,\mathrm{\mathrm{FBL}}\,}}[E]$ in [7, Section 2] defines it as a certain sublattice of positively homogeneous, real-valued functions defined on $E^*$. Since such a function is uniquely determined by its action on $B_{E^*}$, we may regard ${{\,\mathrm{\mathrm{FBL}}\,}}[E]$ as a sublattice of ${\mathbb {R}}^{B_{E^*}}$ simply by restricting its elements to $B_{E^*}$. The universal property of ${{\,\mathrm{\mathrm{FBL}}\,}}[E]$ means that there is a unique lattice homomorphism $S:{{\,\mathrm{\mathrm{FBL}}\,}}[E]\rightarrow X$ such that $S(\delta _{x}) = Tx$ for every $x\in E$, and $\Vert S\Vert =1$. (This lattice homomorphism is usually denoted  ${\widehat{T}}$; we use *S* here to avoid confusion with the lattice homomorphism that we seek to construct.)

We observe that ${{\,\mathrm{\mathrm{FBL}}\,}}[E]$ contains $Y_0$ because $\delta _x\in {{\,\mathrm{\mathrm{FBL}}\,}}[E]$ for every $x\in E$ and ${{\,\mathrm{\mathrm{FBL}}\,}}[E]$ is closed under positively homogeneous continuous function calculus because it is a Banach lattice. Hence we may consider the restriction $S_0:Y_0\rightarrow X$ of the lattice homomorphism *S* to $Y_0$. We claim that $S_0$ is bounded with operator norm at most one. To verify this, we observe that $\nu (f) = \Vert S_0f\Vert$ defines a lattice seminorm $\nu$ on $Y_0$ which satisfies (3.5) because

$$\begin{aligned} \nu (\delta _x) = \bigl \Vert S_0(\delta _x)\bigr \Vert =\Vert Tx\Vert \leqslant \Vert x\Vert \end{aligned}$$

for every $x\in E$. To show that $\nu$ is ${\mathcal {D}}$-convex, write ${\mathcal {D}} = ({\mathcal {G}},M,\vartheta )$, and let $m\in {\mathbb {N}}$, $g\in {\mathcal {G}}_m$, and $f_1,\ldots ,f_m\in (Y_0)_+$, where we assume that $f_1,\ldots ,f_m$ are pairwise disjoint if $\vartheta (g)=0$; note that in that case $S_0f_1,\ldots ,S_0f_m$ are also pairwise disjoint. Therefore, using Corollary 2.3 and the ${\mathcal {D}}$-convexity of *X*, we obtain

$$\begin{aligned} \nu \bigl (g(f_1,\ldots ,f_m)\bigr )&= \Bigl \Vert S_0\bigl (g(f_1,\ldots ,f_m)\bigr )\Bigr \Vert =\bigl \Vert g(S_0f_1,\ldots ,S_0f_m)\bigr \Vert \\&\leqslant M(g)\cdot g\bigl (\Vert S_0f_1\Vert ,\ldots ,\Vert S_0f_m\Vert \bigr ) = M(g)\cdot g\bigl (\nu (f_1),\ldots ,\nu (f_m)\bigr ). \end{aligned}$$

It follows that $\nu \in {\mathcal {N}}_{{\mathcal {D}}}$, and therefore $\Vert f\Vert _{{\mathcal {D}}}\geqslant \nu (f)=\Vert S_0f\Vert$ for every $f\in Y_0$, which proves the claim.

Hence $S_0$ extends uniquely to a lattice homomorphism ${\widehat{T}}:{{\,\mathrm{\mathrm{FBL}}\,}}^{{\mathcal {D}}}[E]\rightarrow X$, and $\Vert {\widehat{T}}\Vert =\Vert S_0\Vert \leqslant 1$. We have ${\widehat{T}}\circ \phi _E^{{\mathcal {D}}}=T$ because ${\widehat{T}}(\delta _x)=S_0(\delta _x)=Tx$ for every $x\in E$. This implies in particular that $\Vert {\widehat{T}}\Vert \geqslant \Vert T\Vert =1$, so $\Vert {\widehat{T}}\Vert = 1$.

Finally, to prove the uniqueness of ${\widehat{T}}$, suppose that $U:{{\,\mathrm{\mathrm{FBL}}\,}}^{{\mathcal {D}}}[E]\rightarrow X$ is any lattice homomorphism satisfying $U(\delta _x) = Tx$ for every $x\in E$. Then Corollary 2.3 implies that

$$\begin{aligned} U\bigl (h(\delta _{x_1},\ldots ,\delta _{x_m})\bigr )&= h\bigl (U(\delta _{x_1}),\ldots ,U(\delta _{x_m})\bigr )\\&= h(Tx_1,\ldots ,Tx_m) ={\widehat{T}}\bigl (h(\delta _{x_1},\ldots ,\delta _{x_m})\bigr ) \end{aligned}$$

for every $m\in {\mathbb {N}}$, $h\in {\mathcal {H}}_m$, and $x_1,\ldots ,x_m\in E$, so *U* and ${\widehat{T}}$ agree on $Y_0$. Since $Y_0$ is dense in ${{\,\mathrm{\mathrm{FBL}}\,}}^{{\mathcal {D}}}[E]$ and *U* and ${\widehat{T}}$ are bounded, we conclude that $U={\widehat{T}}$. $\square$

### Remark 3.4

It follows from general principles that the pair $\bigl ({{\,\mathrm{\mathrm{FBL}}\,}}^{\mathcal {D}}[E], \phi _E^{\mathcal {D}}\bigr )$ constructed in Theorem 3.3 is essentially unique.

### Corollary 3.5

Let *E* be a Banach space and ${\mathcal {D}}$ a convexity condition.

(i) The sublattice generated by the set $\{\delta _{x}\,:\,x\in E\}$ is dense in ${{\,\mathrm{\mathrm{FBL}}\,}}^{{\mathcal {D}}}[E]$.
(ii) Suppose that $S,T:{{\,\mathrm{\mathrm{FBL}}\,}}^{{\mathcal {D}}}[E]\rightarrow X$ are lattice homomorphisms into a Banach lattice *X* satisfying $S\circ \phi _E^{\mathcal {D}} = T\circ \phi _E^{\mathcal {D}}$. Then $S=T$.
(iii) ${{\,\mathrm{\mathrm{FBL}}\,}}^{\mathcal {D}}[E]$ is separable if and only if *E* is separable.

### Proof

(i) The closure of the sublattice of ${{\,\mathrm{\mathrm{FBL}}\,}}^{{\mathcal {D}}}[E]$ generated by $\{\delta _{x}\,:\,x\in E\}$ is a Banach lattice and thus closed under positively homogeneous continuous function calculus. Hence it contains the sublattice $Y_0$ defined by (3.4). This proves the claim because $Y_0$ is dense in ${{\,\mathrm{\mathrm{FBL}}\,}}^{{\mathcal {D}}}[E]$ by definition.
(ii) This is immediate from (i) because lattice homomorphisms are automatically bounded.
(iii) This follows by combining (i) with [3, p. 204, Exercise 9].$\square$

### Example 3.6

Let $p\in [1,\infty ]$. Taking ${\mathcal {D}}=({\mathcal {G}}^p,M,\vartheta )$ for ${\mathcal {G}}^p=\bigl \{\Vert \,\cdot \,\Vert _{\ell _p^m} \,:\,m\in {\mathbb {N}}\bigr \}$, $M\equiv C\in [1,\infty )$ and $\vartheta \equiv 1$ gives the ***free*** *p*-***convex Banach lattice with*** *p*-***convexity constant*** *C* (cf. Remark 3.1). For $C=1$, we denote this space by ${{\,\mathrm{\mathrm{FBL}}\,}}^{(p)}[E]$ and observe that, together with the map $\phi _E = \phi _E^{{\mathcal {D}}}$, it has the properties stated in Theorem 1.1 because every *p*-convex Banach lattice *X* can be renormed to have *p*-convexity constant 1, with the new norm being $M^{(p)}(X)$-equivalent to the original norm. An explicit description of ${{\,\mathrm{\mathrm{FBL}}\,}}^{(p)}[E]$ and its norm will be given in Sect. 6.

Taking instead $\vartheta \equiv 0$ (and ${\mathcal {G}}^p$ and $M\equiv C\in [1,\infty )$ as above), we obtain the ***free Banach lattice satisfying upper*** *p*-***estimates with constant*** *C*.

## Basic properties of ${{\,\mathrm{\mathrm{FBL}}\,}}^{\mathcal {D}}[E]$

The aim of this section is to establish some basic properties of ${{\,\mathrm{\mathrm{FBL}}\,}}^{\mathcal {D}}[E]$. Throughout, ${\mathcal {D}} = ({\mathcal {G}},M,\vartheta )$ denotes a convexity condition, and in situations where no confusion can arise, we will write $\phi _E$ for the canonical map $\phi _E^{\mathcal {D}}:E\rightarrow {{\,\mathrm{\mathrm{FBL}}\,}}^{\mathcal {D}}[E]$.

### Complementation

We begin with a generalization of [7, Corollary 2.7]. Recall that a Banach space *F* is *C*-isomorphic to a complemented subspace of a Banach space *E* for some constant $C\geqslant 1$ if there are operators $U:F\rightarrow E$ and $V:E\rightarrow F$ such that $I_F = V\circ U$ and $\Vert U\Vert \,\Vert V\Vert \leqslant C$, where $I_F$ denotes the identity operator on *F*. In the case where *E* and *F* are Banach lattices, we say that *F* is *C*-***lattice complemented in*** *E* if the operators *U* and *V* can be chosen to be lattice homomorphisms. Note that the condition $I_F = V\circ U$ implies that $P:=U\circ V$ is idempotent, and *U* is an isomorphism of *F* onto the range of *P*.

#### Proposition 4.1

Let *E* and *F* be Banach spaces, where *F* is *C*-isomorphic to a complemented subspace of *E* for some constant $C\geqslant 1$. Then ${{\,\mathrm{\mathrm{FBL}}\,}}^{{\mathcal {D}}}[F]$ is *C*-lattice complemented in ${{\,\mathrm{\mathrm{FBL}}\,}}^{{\mathcal {D}}}[E]$.

#### Proof

Let $U:F\rightarrow E$ and $V:E\rightarrow F$ be operators such that $I_F = V\circ U$ and $\Vert U\Vert \,\Vert V\Vert \leqslant C$. Since $\phi _E\circ U:F\rightarrow {{\,\mathrm{\mathrm{FBL}}\,}}^{{\mathcal {D}}}[E]$ is an operator into a ${\mathcal {D}}$-convex Banach lattice, there is a unique lattice homomorphism ${\widetilde{U}}:=\widehat{\phi _E\circ U}:{{\,\mathrm{\mathrm{FBL}}\,}}^{{\mathcal {D}}}[F]\rightarrow {{\,\mathrm{\mathrm{FBL}}\,}}^{{\mathcal {D}}}[E]$ such that ${\widetilde{U}}\circ \phi _F = \phi _E\circ U$, and $\Vert {\widetilde{U}}\Vert =\Vert \phi _E\circ U\Vert =\Vert U\Vert$ (the last equality follows because $\phi _E$ is an isometry). Similarly we obtain a unique lattice homomorphism ${\widetilde{V}}:=\widehat{\phi _F\circ V}:{{\,\mathrm{\mathrm{FBL}}\,}}^{{\mathcal {D}}}[E]\rightarrow {{\,\mathrm{\mathrm{FBL}}\,}}^{{\mathcal {D}}}[F]$ such that ${\widetilde{V}}\circ \phi _E = \phi _F\circ V$, and $\Vert {\widetilde{V}}\Vert =\Vert V\Vert$. Now we check that

$$\begin{aligned} {\widetilde{V}}\circ {\widetilde{U}}\circ \phi _F = {\widetilde{V}}\circ \phi _E\circ U =\phi _F\circ V\circ U = \phi _F, \end{aligned}$$

so ${\widetilde{V}}\circ {\widetilde{U}}=I_{{{\,\mathrm{\mathrm{FBL}}\,}}^{{\mathcal {D}}}[F]}$ by Corollary 3.5(ii). $\square$

We next characterize when $\phi _E(E)$ is complemented in its free space, cf. [7, Corollary 2.5].

#### Proposition 4.2

Let *E* be a Banach space and $C\geqslant 1$. Then *E* is *C*-isomorphic to a complemented subspace of a ${\mathcal {D}}$-convex Banach lattice if and only if $\phi _E (E)$ is *C*-complemented in ${{\,\mathrm{\mathrm{FBL}}\,}}^{\mathcal {D}}[E]$.

#### Proof

Suppose that *E* is *C*-isomorphic to a complemented subspace of a ${\mathcal {D}}$-convex Banach lattice *X*, so that $I_E=V\circ U$ for some operators $U:E\rightarrow X$ and $V:X\rightarrow E$ with $\Vert U\Vert \,\Vert V\Vert \leqslant C$. Then the inclusion map $J:\phi _E(E)\rightarrow {{\,\mathrm{\mathrm{FBL}}\,}}^{\mathcal {D}}[E]$ and the composite operator $W:=\phi _E\circ V\circ {\widehat{U}}:{{\,\mathrm{\mathrm{FBL}}\,}}^{\mathcal {D}}[E]\rightarrow \phi _E(E)$ satisfy $W\circ J=I_{\phi _E (E)}$ and $\Vert W\Vert \,\Vert J\Vert \leqslant C$, so $\phi _E(E)$ is *C*-complemented in ${{\,\mathrm{\mathrm{FBL}}\,}}^{\mathcal {D}}[E]$.

The converse is immediate because *E* is isometric to $\phi _E(E)$ and ${{\,\mathrm{\mathrm{FBL}}\,}}^{\mathcal {D}}[E]$ is a ${\mathcal {D}}$-convex Banach lattice. $\square$

#### Remark 4.3

It is a famous open question whether every complemented subspace of a Banach lattice is isomorphic to a Banach lattice. Proposition 4.2 reduces this to a question about free Banach lattices, and extends the question to the ${\mathcal {D}}$-convex case.

### Projectivity

We shall next study the projective objects in the category of ${\mathcal {D}}$-convex Banach lattices, beginning with a result which recovers and extends one of the main results of [14].

In line with general conventions, we say that a Banach lattice *Z* is ***projectively universal for the class of separable,*** ${\mathcal {D}}$-***convex Banach lattices*** if *Z* is separable and ${\mathcal {D}}$-convex, and every separable, ${\mathcal {D}}$-convex Banach lattice *X* is lattice isometric to a quotient of *Z*. Note that this is equivalent to the existence of a lattice homomorphism from *Z* onto *X* which maps the open unit ball of *Z* onto the open unit ball of *X*.

#### Theorem 4.4

The Banach lattice ${{\,\mathrm{\mathrm{FBL}}\,}}^{{\mathcal {D}}}[\ell _1]$ is projectively universal for the class of separable, ${\mathcal {D}}$-convex Banach lattices.

#### Proof

Let *X* be a separable, ${\mathcal {D}}$-convex Banach lattice. Using the separable projective universality of $\ell _1$, we can find a linear surjection $T:\ell _1\rightarrow X$ which maps the open unit ball of $\ell _1$ onto the open unit ball of *X*, and hence the induced lattice homomorphism ${\widehat{T}}:{{\,\mathrm{\mathrm{FBL}}\,}}^{{\mathcal {D}}}[\ell _1]\rightarrow X$ maps the open unit ball of ${{\,\mathrm{\mathrm{FBL}}\,}}^{{\mathcal {D}}}[\ell _1]$ onto the open unit ball of *X*. That ${{\,\mathrm{\mathrm{FBL}}\,}}^{{\mathcal {D}}}[\ell _1]$ is separable follows from Corollary 3.5(iii). $\square$

#### Remark 4.5

In Theorem 4.4, one can of course replace $\ell _1$ with any separable Banach space which has every separable, ${\mathcal {D}}$-convex Banach lattice as a quotient. For example, one can iterate the process to get that ${{\,\mathrm{\mathrm{FBL}}\,}}\bigl [{{\,\mathrm{\mathrm{FBL}}\,}}[\ell _1]\bigr ]$ is projectively universal for the class of separable Banach lattices.

#### Remark 4.6

Similar arguments establish an analogous result for arbitrary density character $\kappa$, replacing $\ell _1$ with $\ell _1(\kappa )$.

We present the next simple lemma due to its relevance to Theorem 4.4, and because it will be needed for subsequent results. It shows that the separable, ${\mathcal {D}}$-convex Banach lattices are exactly the lattice quotients of ${{\,\mathrm{\mathrm{FBL}}\,}}^{{\mathcal {D}}}[\ell _1]$.

#### Lemma 4.7

A quotient of a ${\mathcal {D}}$-convex Banach lattice by a closed ideal is ${\mathcal {D}}$-convex.

#### Proof

Let *J* be a closed ideal of a ${\mathcal {D}}$-convex Banach lattice *X*, and denote the quotient homomorphism by $Q:X\rightarrow X/J$. Further, let $g\in {\mathcal {G}}_m$ for some $m\in {\mathbb {N}}$, and take $\varphi _1,\dots ,\varphi _m\in (X/J)_+$, where we suppose that $\varphi _1,\dots ,\varphi _m$ are pairwise disjoint if $\vartheta (g)=0$. For $\varepsilon >0$, we can choose $x_1,\ldots ,x_m\in X_+$ such that $\Vert x_k\Vert \leqslant (1+\varepsilon )\Vert \varphi _k\Vert$ and $Qx_k=\varphi _k$ for each *k*, and we can also arrange that $x_1,\ldots ,x_m$ are pairwise disjoint if $\vartheta (g)=0$ (cf. [17, Section 9]). Then, using Corollary 2.3, we have

$$\begin{aligned}&\bigl \Vert g(\varphi _1,\dots ,\varphi _m)\bigr \Vert =\Bigl \Vert Q\bigl (g(x_1,\dots ,x_m)\bigr )\Bigr \Vert \leqslant M(g)\cdot g\bigl (\Vert x_1\Vert ,\dots ,\Vert x_m\Vert \bigr )\\&\qquad \quad \leqslant M(g)\cdot g\Bigl ((1+\varepsilon )\Vert \varphi _1\Vert , \dots ,(1+\varepsilon )\Vert \varphi _m\Vert \Bigr ) \xrightarrow {\varepsilon \rightarrow 0} M(g)\cdot g\bigl (\Vert \varphi _1\Vert ,\dots ,\Vert \varphi _m\Vert \bigr ), \end{aligned}$$

and the conclusion follows. $\square$

The notion of a ***projective*** Banach lattice was introduced in [17]. Informally, a Banach lattice *P* is projective if every lattice homomorphism from *P* to a quotient of a Banach lattice *X* can be lifted to a lattice homomorphism into *X*, with control of the norm. As a consequence of the fact that $\ell _1(A)$ is a projective Banach space for any nonempty set *A*, it was shown in [17] that ${{\,\mathrm{\mathrm{FBL}}\,}}\bigl [\ell _1(A)\bigr ]$ is a projective Banach lattice. Other examples of projective Banach lattices include all finite dimensional Banach lattices [17] and *C*(*K*) spaces for every compact Hausdorff space *K* which is an absolute neighbourhood retract; see [4, 17], as well as [5] for more recent related results.

To conclude this section, we find a Banach space property of *E* which characterizes when ${{\,\mathrm{\mathrm{FBL}}\,}}[E]$ is projective. Note in this connection that it was shown in [4] that if ${{\,\mathrm{\mathrm{FBL}}\,}}[E]$ is projective, then necessarily *E* has the Schur property. Since we now also have the spaces ${{\,\mathrm{\mathrm{FBL}}\,}}^{\mathcal {D}}[E]$ — and our characterizations extend to these spaces in the appropriate way — we introduce two new definitions. Part (i) extends the definition of projectivity in [17], while (ii) is a variant that makes sense for arbitrary Banach spaces.

#### Definition 4.8

(i) A Banach lattice *P* is ***projective for*** ${\mathcal {D}}$-***convex Banach lattices*** if, for every closed ideal *J* of a ${\mathcal {D}}$-convex Banach lattice *X*, every lattice homomorphism $T:P\rightarrow X/J$, and every $\varepsilon >0$, there is a lattice homomorphism ${\widehat{T}}:P\rightarrow X$ such that $T=Q\circ {\widehat{T}}$ and $\Vert {\widehat{T}}\Vert \leqslant (1+\varepsilon )\Vert T\Vert$, where $Q:X\rightarrow X/J$ denotes the quotient homomorphism.
(ii) A Banach space *E* is ***linearly projective for*** ${\mathcal {D}}$-***convex Banach lattices*** if, for every closed ideal *J* of a ${\mathcal {D}}$-convex Banach lattice *X*, every operator $T:E\rightarrow X/J$, and every $\varepsilon >0$, there is an operator ${\widehat{T}}:E\rightarrow X$ such that $T=Q\circ {\widehat{T}}$ and $\Vert {\widehat{T}}\Vert \leqslant (1+\varepsilon )\Vert T\Vert$, again with $Q:X\rightarrow X/J$ denoting the quotient homomorphism.

Note that in the above definitions we require that *X* is ${\mathcal {D}}$-convex and this implies that *X*/*J* is as well, by Lemma 4.7. For convenience, given convexity conditions ${\mathcal {D}}$ and ${\mathcal {D}}'$, let us write ${\mathcal {D}}'\leqslant {\mathcal {D}}$ whenever ${\mathcal {D}}$-convexity implies $\mathcal {D'}$-convexity. The following result (applied with ${\mathcal {D}}={\mathcal {D}}'$) clarifies the relationship between these two notions, as well as a third “hybrid” notion.

#### Proposition 4.9

Suppose that ${\mathcal {D}}$ and ${\mathcal {D}}'$ are convexity conditions with ${\mathcal {D}}'\leqslant {\mathcal {D}}$. Then the following three conditions are equivalent for a Banach space$E:$

(i) *E* is linearly projective for ${\mathcal {D}}$-convex Banach lattices.
(ii) ${{\,\mathrm{\mathrm{FBL}}\,}}^\mathcal {D'}[E]$ is projective for ${\mathcal {D}}$-convex Banach lattices.
(iii) For every closed ideal *J* of a ${\mathcal {D}}$-convex Banach lattice *X*,  every lattice homomorphism $T:{{\,\mathrm{\mathrm{FBL}}\,}}^\mathcal {D'}[E]\rightarrow X/J,$ and every $\varepsilon >0,$ there is an operator ${\widehat{T}}:{{\,\mathrm{\mathrm{FBL}}\,}}^\mathcal {D'}[E]\rightarrow X$ such that $T=Q\circ {\widehat{T}}$ and $\Vert {\widehat{T}}\Vert \leqslant (1+\varepsilon )\Vert T\Vert ,$ where $Q:X\rightarrow X/J$ denotes the quotient homomorphism.

#### Proof

(i) $\Rightarrow$ (ii) Suppose that *E* is linearly projective for ${\mathcal {D}}$-convex Banach lattices, and let $T:{{\,\mathrm{\mathrm{FBL}}\,}}^\mathcal {D'}[E]\rightarrow X/J$ be a lattice homomorphism, where *J* is a closed ideal of a ${\mathcal {D}}$-convex Banach lattice *X*. By the hypothesis, for every $\varepsilon >0$, we can lift the operator $S:=T\circ \phi _E^{{\mathcal {D}}'}:E \rightarrow X/J$ to an operator ${\widehat{S}}:E\rightarrow X$ with $Q\circ {\widehat{S}}=S$ and $\Vert {\widehat{S}}\Vert \leqslant (1+\varepsilon )\Vert S\Vert$, where $Q :X\rightarrow X/J$ is the quotient homomorphism. Since *X* is $\mathcal {D'}$-convex,Theorem 3.3 implies that ${\widehat{S}}$ lifts to a lattice homomorphism ${\widehat{T}}:{{\,\mathrm{\mathrm{FBL}}\,}}^\mathcal {D'}[E]\rightarrow X$ with ${\widehat{T}}\circ \phi ^{{\mathcal {D}}'}_E = {\widehat{S}}$ and $\Vert {\widehat{T}}\Vert =\Vert {\widehat{S}}\Vert$. We check that ${\widehat{T}}$ has the required properties: $Q\circ {\widehat{T}} = T$ by Corollary 3.5(ii) because

$$\begin{aligned} Q\circ {\widehat{T}}\circ \phi _E^{{\mathcal {D}}'} =Q\circ {\widehat{S}} = S =T\circ \phi _E^{{\mathcal {D}}'}, \end{aligned}$$

and

$$\begin{aligned} \Vert {\widehat{T}}\Vert \leqslant (1+\varepsilon )\Vert S\Vert =(1+\varepsilon )\bigl \Vert T\circ \phi _E^{{\mathcal {D}}'}\bigr \Vert \leqslant (1+\varepsilon )\Vert T\Vert . \end{aligned}$$

(ii) $\Rightarrow$ (iii) is obvious.

(iii) $\Rightarrow$ (i) Suppose that (iii) is satisfied, and let $T:E\rightarrow X/J$ be an operator, where *J* is a closed ideal of a ${\mathcal {D}}$-convex Banach lattice *X*. Using Lemma 4.7 and Theorem 3.3, we can find a lattice homomorphism $S:{{\,\mathrm{\mathrm{FBL}}\,}}^\mathcal {D'}[E]\rightarrow X/J$ with $S\circ \phi _E^{{\mathcal {D}}'} = T$ and $\Vert S\Vert =\Vert T\Vert$. By (iii), for every $\varepsilon >0$, there is an operator ${\widehat{S}}:{{\,\mathrm{\mathrm{FBL}}\,}}^\mathcal {D'}[E]\rightarrow X$ such that $S=Q\circ {\widehat{S}}$ and $\Vert {\widehat{S}}\Vert \leqslant (1+\varepsilon )\Vert S\Vert$. Then the operator ${\widehat{T}}:={\widehat{S}}\circ \phi _E^{{\mathcal {D}}'}:E\rightarrow X$ has the desired properties: Its norm is at most $(1+\varepsilon )\Vert T\Vert$ and

$$\begin{aligned} Q\circ {\widehat{T}} = Q\circ {\widehat{S}}\circ \phi _E^{{\mathcal {D}}'} = S\circ \phi _E^{{\mathcal {D}}'} = T. \end{aligned}$$

$$\square $$

#### Remark 4.10

One may now wonder when ${{\,\mathrm{\mathrm{FBL}}\,}}[E]$ is linearly projective for Banach lattices. This will essentially never happen. Indeed, if it were then ${{\,\mathrm{\mathrm{FBL}}\,}}[E]$ would have the Schur property, so in particular would be order continuous. However, ${{\,\mathrm{\mathrm{FBL}}\,}}[E]$ will not be order continuous as long as $\dim E>1$. It is also not true that linear projectivity implies lattice projectivity: $\ell _1(A)$ is a linearly projective Banach space, but it follows from [17, Corollary 10.5] that it is not a projective Banach lattice when *A* is uncountable.

## Free AM-spaces

An ***AM-space*** is a Banach lattice *X* for which $\Vert x\vee y\Vert =\Vert x\Vert \vee \Vert y\Vert$ whenever $x,y\in X_+$ are disjoint. A ***unital AM-space*** is a nonzero AM-space *X* which contains a positive element *e* such that $I_e=X$ and the norm $\Vert \,\cdot \,\Vert _e$ defined by (2.2) is equal to the given norm on *X*.

Kakutani’s famous representation theorem for AM-spaces states that a Banach lattice is an AM-space if and only if it admits an isometric lattice homomorphism into *C*(*K*) for some compact Hausdorff space *K*, and it is a unital AM-space if and only if it is isometrically lattice isomorphic to *C*(*K*) for some *K* (see, e.g., [15, Theorem 1.b.6]).

We begin this section by identifying the convexity conditions ${\mathcal {D}}$ which correspond to AM-spaces, and we then show that, for a given Banach space *E*, they all give rise to the same ${\mathcal {D}}$-convex free Banach lattice. For the avoidance of any doubt in the following definition, recall that $\bigl \Vert (s,t)\bigr \Vert _{\ell _\infty ^2} = |s|\vee |t|$ for $s,t\in {\mathbb {R}}$.

### Definition 5.1

An ***AM-convexity condition*** is a convexity condition ${\mathcal {D}} = ({\mathcal {G}},M,\vartheta )$ for which $\Vert \,\cdot \,\Vert _{\ell _\infty ^2}\in {\mathcal {G}}$ with $M(\Vert \,\cdot \,\Vert _{\ell _\infty ^2})=1$.

### Lemma 5.2

The following conditions are equivalent for a Banach lattice $X:$

(i) *X* is an AM-space.
(ii) *X* is ${\mathcal {D}}$-convex for every convexity condition ${\mathcal {D}}$.
(iii) *X* is ${\mathcal {D}}$-convex for some AM-convexity condition ${\mathcal {D}}$.

### Proof

(i) $\Rightarrow$ (ii): Suppose that *X* is an AM-space, and take an isometric lattice homomorphism $T:X\rightarrow C(K)$ for some compact Hausdorff space *K*. Let $m\in {\mathbb {N}}$, $g\in {\mathcal {H}}_m^{>0}$, and $x_1,\ldots ,x_m\in X_+$. Since $0\leqslant (Tx_j)(t)\leqslant \Vert Tx_j\Vert _\infty =\Vert x_j\Vert$ for every $t\in K$ and $j=1,\ldots ,m$, we have

$$\begin{aligned} g\bigl (\Vert x_1\Vert ,\ldots ,\Vert x_m\Vert \bigr ) \geqslant g\bigl ((Tx_1)(t),\ldots ,(Tx_m)(t)\bigr ) = T\bigl (g(x_1,\ldots ,x_m)\bigr )(t)\geqslant 0, \end{aligned}$$

where the equality follows from Corollary 2.3 and the fact that the positively homogeneous continuous function calculus is defined pointwise in *C*(*K*). Taking the supremum over all $t\in K$, we obtain

$$\begin{aligned} g\bigl (\Vert x_1\Vert ,\ldots ,\Vert x_m\Vert ) \geqslant \Bigl \Vert T\bigl (g(x_1,\ldots ,x_m)\bigr )\Bigr \Vert _\infty =\bigl \Vert g(x_1,\ldots ,x_m)\bigr \Vert , \end{aligned}$$

which shows that (3.2) is satisfied because $M(g)\geqslant 1$. Hence *X* is ${\mathcal {D}}$-convex, no matter which convexity condition ${\mathcal {D}}$ we consider.

(ii) $\Rightarrow$ (iii) is trivial.

(iii) $\Rightarrow$ (i) Suppose that *X* is ${\mathcal {D}}$-convex for some AM-convexity condition ${\mathcal {D}}$. Then we have $\Vert x\vee y\Vert \leqslant \Vert x\Vert \vee \Vert y\Vert$ whenever $x,y\in X_+$ are disjoint. The opposite inequality is always true by monotonicity of the norm, so *X* is an AM-space. $\square$

Let *E* be a Banach space and ${\mathcal {D}}$ a convexity condition. Corollary 3.5(i) shows that we may view ${{\,\mathrm{\mathrm{FBL}}\,}}^{\mathcal {D}}[E]$ as the completion of the sublattice *L* of ${\mathbb {R}}^{B_{E^*}}$ generated by the set $\{\delta _{x}\,:\,x\in E\}$ with respect to the norm $\Vert \,\cdot \,\Vert _{{\mathcal {D}}}$ given by (3.6). In fact $L\subseteq C(B_{E^*})$, where $B_{E^*}$ is equipped with the relative weak$^*$ topology, because $\delta _x$ is weak$^*$ continuous for every $x\in E$, and using the seminorms $\nu _{x^*}$ defined by (3.7) for $x^*\in B_{E^*}$, we see that $\Vert f\Vert _\infty \leqslant \Vert f\Vert _{\mathcal {D}}$ for every $f\in L$. Hence the inclusion map

$$\begin{aligned} \bigl (L,\Vert \,\cdot \,\Vert _{{\mathcal {D}}}\bigr )\rightarrow \bigl ({\overline{L}}^{\Vert \,\cdot \,\Vert _\infty },\Vert \,\cdot \,\Vert _\infty \bigr ) \end{aligned}$$

extends to a lattice homomorphism of norm at most 1 defined on ${{\,\mathrm{\mathrm{FBL}}\,}}^{{\mathcal {D}}}[E]$. Despite this, it is not clear if this map is injective — we do not even know this for the free Banach lattice satisfying an upper *p*-estimate with constant 1. It is, however, an isometric isomorphism provided that ${\mathcal {D}}$ is an AM-convexity condition, as we shall prove next. For that reason, we term ${\overline{L}}^{\Vert \,\cdot \,\Vert _\infty }$ the ***free AM-space over*** *E*.

### Theorem 5.3

Let *E* be a Banach space and ${\mathcal {D}}$ an AM-convexity condition. Then ${{\,\mathrm{\mathrm{FBL}}\,}}^{{\mathcal {D}}}[E]$ is isometrically lattice isomorphic to the $\Vert \,\cdot \,\Vert _\infty$-closed sublattice of $C(B_{E^*})$ generated by $\{\delta _{x}\,:\,x\in E\}$.

### Proof

By the above remarks, it suffices to show that $\Vert f\Vert _\infty \geqslant \Vert f\Vert _{\mathcal {D}}$ for every $f\in L$. Write *f* as $f=\bigvee _{j=1}^n\delta _{x_j}-\bigvee _{j=1}^n\delta _{y_j}$, where $n\in {\mathbb {N}}$ and $x_1,\ldots ,x_n,y_1,\ldots ,y_n\in E$, using (2.1). Since ${{\,\mathrm{\mathrm{FBL}}\,}}^{{\mathcal {D}}}[E]$ is ${\mathcal {D}}$-convex, Lemma 5.2 implies that it is an AM-space, so we can find an isometric lattice homomorphism $U:{{\,\mathrm{\mathrm{FBL}}\,}}^{{\mathcal {D}}}[E]\rightarrow C(K)$ for some compact Hausdorff space *K*. For $t\in K$, let $\eta _t\in B_{C(K)^*}$ be the evaluation functional at *t*, and define $x^*=\eta _t\circ U\circ \phi _E^{{\mathcal {D}}}\in B_{E^*}$. Then we have

$$\begin{aligned} \delta _x(x^*) =(\eta _t\circ U\circ \phi _E^{{\mathcal {D}}})(x) = (U\delta _x)(t) \end{aligned}$$

for every $x\in E$, so that

$$\begin{aligned} f(x^*) =\bigvee _{j=1}^n\delta _{x_j}(x^*) - \bigvee _{j=1}^n\delta _{y_j}(x^*) =\bigvee _{j=1}^n(U\delta _{x_j})(t) - \bigvee _{j=1}^n(U\delta _{y_j})(t) = (Uf)(t). \end{aligned}$$

It follows that

$$\begin{aligned} \Vert f\Vert _\infty \geqslant \sup _{t\in K}|(Uf)(t)|=\Vert Uf\Vert _\infty =\Vert f\Vert _{{\mathcal {D}}}, \end{aligned}$$

as required. $$\square $$

Our next result complements Theorem 5.3 by identifying the free unital AM-space over a Banach space *E*. More precisely, in the light of Kakutani’s representation theorem for unital AM-spaces stated above, it can be paraphrased as saying that the pair $\bigl (C(B_{E^*}),\phi _E\bigr )$ is the free unital AM-space over *E*, where $B_{E^*}$ is equipped with the relative weak$^*$ topology and $\phi _E:E\rightarrow C(B_{E^*})$ denotes the linear isometry given by $\phi _E(x) = \delta _x$, as usual.

### Theorem 5.4

Let *E* be a Banach space. For every compact Hausdorff space *K* and every norm one operator $T:E\rightarrow C(K)$, there exists a unique lattice homomorphism ${\widehat{T}}:C(B_{E^*})\rightarrow C(K)$ such that ${\widehat{T}}\circ \phi _E=T$ and ${\widehat{T}}\mathbb {1}=\mathbb {1}$, where $\mathbb {1}$ denotes the constant function 1. Moreover, ${\widehat{T}}$ is an algebra homomorphism with $\Vert {\widehat{T}}\Vert =1$.

### Proof

Since $\Vert T\Vert =1$, the map $t\mapsto \eta _t\circ T$, where $\eta _t$ is the evaluation functional at *t*, maps *K* into $B_{E^*}$, and it is continuous with respect to the relative weak$^*$ topology on $B_{E^*}$, so we can define a map ${\widehat{T}}:C(B_{E^*})\rightarrow C(K)$ by ${\widehat{T}}(f)(t) = f(\eta _t\circ T)$ for $f\in C(B_{E^*})$ and $t\in K$. Since the algebraic and lattice operations in both $C(B_{E^*})$ and *C*(*K*) are defined pointwise, it is easy to check that ${\widehat{T}}$ is a lattice and algebra homomorphism with ${\widehat{T}}\mathbb {1}=\mathbb {1}$ (see also [16, Theorem 3.2.12] for a more global picture of these maps). Moreover, we have

$$\begin{aligned} \bigl ({\widehat{T}}\circ \phi _E\bigr )(x)(t) =\delta _x(\eta _t\circ T)=(\eta _t\circ T)(x) = (Tx)(t) \end{aligned}$$

for every $x\in E$ and $t\in K$, so that ${\widehat{T}}\circ \phi _E = T$. This implies in particular that $\Vert {\widehat{T}}\Vert \geqslant \Vert T\Vert =1$. On the other hand, $\bigl |({\widehat{T}}f)(t)\bigr |= \bigl |f(\eta _t\circ T)\bigr |\leqslant \Vert f\Vert _\infty$ for every $t\in K$ and $f\in C(B_{E^*})$, so that $\Vert {\widehat{T}}f\Vert _\infty \leqslant \Vert f\Vert _\infty$, and therefore $\Vert {\widehat{T}}\Vert =1$.

Finally, to prove uniqueness, suppose that $U:C(B_{E^*})\rightarrow C(K)$ is any lattice homomorphism satisfying $U\circ \phi _E=T$ and $U\mathbb {1}=\mathbb {1}$. Then ${\widehat{T}}$ and *U* agree on the sublattice of $C(B_{E^*})$ generated by $\{\delta _x \,:\,x\in E\}\cup \{\mathbb {1}\}$. The Stone–Weierstrass Theorem implies that this sublattice is dense in $C(B_{E^*})$, and therefore, being bounded, ${\widehat{T}}$ and *U* are equal. $\square$

## An explicit formula for the norm of the free p-convex Banach lattice

The aim of this section is to verify the explicit formula (1.1) for the norm of the free *p*-convex Banach lattice ${{\,\mathrm{\mathrm{FBL}}\,}}^{(p)}[E]$. Throughout, $p\in (1,\infty )$, *E* is a Banach space, *H*[*E*] denotes the vector lattice of all positively homogeneous functions $E^*\rightarrow {\mathbb {R}}$, $\Vert f\Vert _p$ is defined by (1.1) for every $f\in H[E]$, and *L* denotes the sublattice of *H*[*E*] generated by the evaluation maps $\delta _x$ for $x\in E$. Note that this definition of *L* differs slightly from the one we used in the previous section, where the functions in *L* were defined on $B_{E^*}$, not $E^*$. However, as already remarked in the proof of Theorem 3.3, this difference is purely formal because a positively homogeneous function $E^*\rightarrow {\mathbb {R}}$ is uniquely determined by its action on $B_{E^*}$.

To simplify notation, we write

$$\begin{aligned} \mu _{p}(x_1^*,\dots ,x_n^*) =\sup _{x\in B_E}\Bigl (\sum _{k=1}^n |x_k^*(x)|^p\Bigr )^{\frac{1}{p}} \end{aligned}$$

for the weak *p*-summing norm of a finite sequence $(x_k^*)_{k=1}^n$ in $E^*$.

Let us begin by trying to motivate the expression (1.1) for the norm of ${{\,\mathrm{\mathrm{FBL}}\,}}^{(p)}[E]$. Consider an operator $T:E\rightarrow \ell _p^n$ for some $n\in \mathbb N$. Writing $(e_k)_{k=1}^n$ for the unit vector basis of $\ell _p^n$, we can express *T* as

$$\begin{aligned} T(x)=\sum _{k=1}^n x_k^*(x) e_k \end{aligned}$$

for a certain finite sequence $(x_k^*)_{k=1}^n$ in $E^*$ and every $x\in E$, and we have $\Vert T\Vert = \mu _{p}(x_1^*,\ldots ,x_n^*)$ in the notation introduced above. (In fact $x_k^*=T^*e_k^*$, but this formula will not be helpful for our purposes.) It is easy to check that the only way to extend *T* to a lattice homomorphism ${\widehat{T}}:L \rightarrow \ell _p^n$ is by defining

$$\begin{aligned} {\widehat{T}}f=\sum _{k=1}^n f(x_k^*)e_k \end{aligned}$$

for every $f\in L$. Thus, we must have

$$\begin{aligned} \Bigl (\sum _{k=1}^n \bigl |f(x_k^*)\bigr |^p\Bigr )^{\frac{1}{p}} =\Vert {\widehat{T}}f\Vert _{\ell _p^n} \leqslant \Vert {\widehat{T}}\Vert \,\Vert f\Vert _{{{\,\mathrm{\mathrm{FBL}}\,}}^{(p)}[E]} =\mu _{p}(x_1^*,\ldots ,x_n^*)\,\Vert f\Vert _{{{\,\mathrm{\mathrm{FBL}}\,}}^{(p)}[E]}. \end{aligned}$$

Taking the supremum over all possible choices of the operator *T* subject to $\Vert T\Vert \leqslant 1$, we conclude that $\Vert f\Vert _p$ defined by (1.1) satisfies the inequality $\Vert f\Vert _p\leqslant \Vert f\Vert _{{{\,\mathrm{\mathrm{FBL}}\,}}^{(p)}[E]}$. Morally speaking, establishing equality of these two norms means that extending operators into arbitrary *p*-convex Banach lattices can in a certain sense be reduced to the extension of operators into the spaces $\ell _p^n$ for $n\in {\mathbb {N}}$.

We now turn to the explicit description of ${{\,\mathrm{\mathrm{FBL}}\,}}^{(p)}[E]$. It is easy to see that

$$\begin{aligned} H_p[E]:=\bigl \{f\in H[E] \,:\,\Vert f\Vert _{p} <\infty \bigr \} \end{aligned}$$

is a sublattice of *H*[*E*] and that $\Vert \,\cdot \,\Vert _p$ defines a complete *p*-convex lattice norm on $H_p[E]$ with *p*-convexity constant one. Moreover, $\Vert \delta _x\Vert _p=\Vert x\Vert$ for every $x\in E$, so $H_p[E]$ contains *L* as a sublattice. Hence we can define ${{\,\mathrm{\mathrm{FBL}}\,}}_p[E]$ as the closure of *L* in $H_p[E]$, and the map $\phi _E:E\rightarrow {{\,\mathrm{\mathrm{FBL}}\,}}_p[E]$ given by $\phi _E(x)=\delta _x$ is a linear isometry. Note the position of the index *p*: We write ${{\,\mathrm{\mathrm{FBL}}\,}}_p[E]$ for the Banach lattice that we have just defined to distinguish it from the previously defined Banach lattice ${{\,\mathrm{\mathrm{FBL}}\,}}^{(p)}[E]$. However, our next theorem will identify the pair $({{\,\mathrm{\mathrm{FBL}}\,}}_p[E],\phi _E)$ as the free *p*-convex Banach lattice generated by *E*, so once we have proved it, this distinction will no longer be necessary.

### Theorem 6.1

Let *X* be a *p*-convex Banach lattice and $T:E\rightarrow X$ an operator. There is a unique lattice homomorphism ${\widehat{T}}:{{\,\mathrm{\mathrm{FBL}}\,}}_p[E]\rightarrow X$ such that ${\widehat{T}}\circ \phi _E=T,$ and $\Vert {\widehat{T}}\Vert \leqslant M^{(p)}(X)\,\Vert T\Vert ,$ where $M^{(p)}(X)$ denotes the *p*-convexity constant of *X*.

### Proof

As in the proof of [7, Theorem 2.5], there is a unique lattice homomorphism ${\widehat{T}}:L\rightarrow X$ such that ${\widehat{T}}(\delta _x) = Tx$ for every $x\in E$. Our objective is to show that

6.1 $$\begin{aligned} \Vert {{\widehat{T}}} f\Vert _{X}\leqslant M^{(p)}(X)\,\Vert T\Vert \,\Vert f\Vert _p \end{aligned}$$

for every $f\in L$, as this will ensure that ${\widehat{T}}$ extends uniquely to a lattice homomorphism defined on all of ${{\,\mathrm{\mathrm{FBL}}\,}}_p[E]$, and the extension has norm at most $M^{(p)}(X)\,\Vert T\Vert$.

We split the proof of the inequality (6.1) in two parts: First we establish it in the special case where $X=L_p(\mu )$ for some measure space $(\Omega ,\Sigma ,\mu )$, and then we show how to deduce the general version from the special case.

Thus, suppose first that $X=L_p(\mu )$ for some measure space $(\Omega ,\Sigma ,\mu )$, and let $f\in L$. By (2.1), we can write $f=\bigvee _{i=1}^n \delta _{x_i}-\bigvee _{j=1}^n \delta _{y_j}$ for some $n\in {\mathbb {N}}$ and $(x_i)_{i=1}^n$, $(y_j)_{j=1}^n$ in *E*. Consider the family of sets $(A_{ij})_{i,j=1}^n\subset \Sigma$ defined by

$$\begin{aligned} A_{ij} =\biggl \{\omega \in \Omega \,:\,\bigvee _{k=1}^n Tx_k(\omega )=Tx_i(\omega ),\ \bigvee _{l=1}^n Ty_l(\omega )=Ty_j(\omega )\biggr \}. \end{aligned}$$

Clearly $\bigcup _{i,j=1}^n A_{ij}=\Omega$. By a standard disjointification process, replacing $A_{ij}$ with $A_{ij}{\setminus }\bigcup _{(k,l)\prec (i,j)} A_{kl}$, where $\prec$ is any total order on the index set $\bigl \{(i,j)\,:\,1\leqslant i,j\leqslant n\bigr \}$, we may arrange that the sets $(A_{ij})_{i,j=1}^n$ are pairwise disjoint.

For every $1\leqslant i,j\leqslant n$, define

$$\begin{aligned} A_{ij}^+=\bigl \{\omega \in A_{ij}\,:\,T(x_i-y_j)(\omega ) \geqslant 0\bigr \} \quad \text {and}\quad A_{ij}^-=A_{ij}{\setminus } A_{ij}^+, \end{aligned}$$

and choose positive functions $g_{ij},h_{ij}\in L_{p^*}(\mu )=L_p(\mu )^*$, where $p^*\in (1,\infty )$ is the conjugate exponent of *p*, such that $\Vert g_{ij}\Vert _{L_{p^*}} = \Vert h_{ij}\Vert _{L_{p^*}} =1$,

$$\begin{aligned}&\Bigl \Vert T(x_i-y_j)\chi _{A_{ij}^+}\Bigr \Vert _{L_p} =\bigl \langle T(x_i-y_j)\chi _{A_{ij}^+}, g_{ij}\bigr \rangle =\int _{A_{ij}^+}T(x_i-y_j)g_{ij}\,d\mu ,\\ \end{aligned}$$

and

$$\begin{aligned}&\Bigl \Vert T(y_j-x_i)\chi _{A_{ij}^-}\Bigr \Vert _{L_p} =\bigl \langle T(y_j-x_i)\chi _{A_{ij}^-}, h_{ij}\bigr \rangle =\int _{A_{ij}^-}T(y_j-x_i)h_{ij}\,d\mu . \end{aligned}$$

We may without loss of generality assume that $g_{ij}$ and $h_{ij}$ are supported in $A_{ij}^+$ and $A_{ij}^-$, respectively. Then the set $\bigl \{g_{ij},h_{ij}\,:\,1\leqslant i,j\leqslant n\bigr \}$ is 1-equivalent to the unit vector basis of $\ell _{p^*}^{2n^2}$, and consequently the functionals $x_{ij}^*=T^*g_{ij}\in E^*$ and $y_{ij}^*=T^*h_{ij}\in E^*$ satisfy

$$\begin{aligned}&\biggl (\sum _{i,j=1}^n\bigl |x_{ij}^*(x)\bigr |^p+\bigl |y_{ij}^*(x)\bigr |^p\biggr )^{\frac{1}{p}} =\biggl (\sum _{i,j=1}^n \biggl |\int _{A_{ij}^+}(Tx)g_{ij}\,d\mu \biggr |^p +\biggl |\int _{A_{ij}^-}(Tx)h_{ij}\,d\mu \biggr |^p\biggr )^{\frac{1}{p}}\\&\quad =\sup \biggl \{\sum _{i,j=1}^n a_{ij}\int _{A_{ij}^+}(Tx)g_{ij}\,d\mu +b_{ij}\int _{A_{ij}^-}(Tx)h_{ij}\,d\mu \,:\,(a_{ij},b_{ij})_{i,j=1}^n\in B_{\ell _{p^*}^{2n^2}} \biggr \}\\&\quad \leqslant \sup \bigl \{\langle Tx, g\rangle \,:\,g\in B_{L_{p*}(\mu )}\bigr \} =\Vert Tx\Vert _{L_p} \end{aligned}$$

for every $x\in E$. Taking the supremum over $x\in B_E$, we conclude that

6.2 $$\begin{aligned} \mu _{p}(x^*_{11},\ldots ,x^*_{nn},y^*_{11},\ldots ,y^*_{nn}) \leqslant \Vert T\Vert . \end{aligned}$$

Since $g_{ij}$ is positive, the definition of $A^+_{ij}$ yields that

$$\begin{aligned} \bigl |f(x_{ij}^*)\bigr |= & {} \biggl |\bigvee _{k=1}^n\int (Tx_k)g_{ij}\,d\mu -\bigvee _{l=1}^n\int (Ty_l)g_{ij}\,d\mu \biggr |\\= & {} \int T(x_i-y_j)g_{ij}\,d\mu =\bigl \Vert T(x_i-y_j)\chi _{A_{ij}^+}\bigr \Vert _{L_p} =\bigl \Vert ({\widehat{T}} f )\chi _{A_{ij}^+}\bigr \Vert _{L_p} \end{aligned}$$

and similarly $\bigl |f(y_{ij}^*)\bigr |=\bigl \Vert ({\widehat{T}} f )\chi _{A_{ij}^-}\bigr \Vert _{L_p}$ for every $1\leqslant i,j\leqslant n$. Combining (1.1) and (6.2) with these identities, we deduce that

$$\begin{aligned}&\Vert T\Vert \,\Vert f\Vert _p \geqslant \biggl (\sum _{i,j=1}^n \bigl |f(x_{ij}^*)\bigr |^p +\bigl |f(y_{ij}^*)\bigr |^p\biggr )^{\frac{1}{p}}\\&\quad =\biggl (\sum _{i,j=1}^n\bigl \Vert ({\widehat{T}} f) \chi _{A_{ij}^+}\bigr \Vert ^p_{L_p} +\bigl \Vert ({\widehat{T}} f) \chi _{A_{ij}^-}\bigr \Vert ^p_{L_p}\biggr )^{\frac{1}{p}} =\Vert {\widehat{T}} f\Vert _{L_p}, \end{aligned}$$

which establishes (6.1) for $X=L_p(\mu )$ because $M^{(p)}(L_p(\mu ))=1$.

We are now ready to tackle the general case where *X* is an arbitrary *p*-convex Banach lattice. Given $f\in L$, choose $x^*\in X^*_+$ with $\Vert x^*\Vert =1$ and $x^*\bigl (|{\widehat{T}}f|\bigr )=\Vert {\widehat{T}}f\Vert _X$. Let $N_{x^*}$ denote the null ideal generated by $x^*$, that is, $N_{x^*}=\bigl \{x\in X\,:\,x^*\bigl (|x|\bigr )=0\bigr \}$, and let *Y* be the completion of the quotient lattice $X/N_{x^*}$ with respect to the norm $\Vert x+N_{x^*}\Vert :=x^*\bigl (|x|\bigr )$. Since this is an abstract $L_1$-norm, *Y* is lattice isometric to $L_1(\Omega ,\Sigma ,\mu )$ for some measure space $(\Omega ,\Sigma ,\mu )$ (see, e.g., [15, Theorem 1.b.2]). The canonical quotient map of *X* onto $X/N_{x^*}$ induces a lattice homomorphism $Q:X\rightarrow L_1(\Omega ,\Sigma ,\mu )$ with $\Vert Q\Vert =1$. For our purposes, we may without loss of generality assume that $(\Omega ,\Sigma ,\mu )$ is $\sigma$-finite, passing for instance to the band generated by $Q({\widehat{T}} f)$.

Since *Q* is a lattice homomorphism and *X* is *p*-convex, we have

$$\begin{aligned} \biggl \Vert \Bigl (\sum _{k=1}^n\bigl |Q(x_k)\bigr |^p\Bigr )^{\frac{1}{p}}\biggr \Vert _{L_1(\mu )} \leqslant \biggl \Vert \Bigl (\sum _{k=1}^n|x_k|^p\Bigr )^{\frac{1}{p}}\biggr \Vert _X \leqslant M^{(p)}(X)\,\Bigl (\sum _{k=1}^n\Vert x_k\Vert _X^p\Bigr )^{\frac{1}{p}} \end{aligned}$$

for every $n\in {\mathbb {N}}$ and $x_1,\dots , x_n\in X$. Hence the Maurey–Nikishin Factorization Theorem (see, e.g, [1, Theorem 7.1.2.], and recall that $p<\infty$) yields a positive function $h\in L_1(\Omega ,\Sigma ,\mu )$ with $\int _\Omega h\,d\mu =1$ such that *Q* is bounded if we regard it as an operator into $L_p(h\,d\mu )$. More precisely, we have a factorization diagramwhere $Sx=h^{-1}Qx$ satisfies $\Vert S\Vert \leqslant M^{(p)}(X)$ and $j_h(g)=gh$ is an isometric embedding. Note in particular that *S* is also a lattice homomorphism.

Let us now consider the composite operator $R=S\circ T:E\rightarrow L_p(h\,d\mu )$. By the first part of the proof, we know that there is a unique lattice homomorphism ${\widehat{R}}:{{\,\mathrm{\mathrm{FBL}}\,}}_p[E]\rightarrow L_p(h\,d\mu )$ such that ${\widehat{R}}(\delta _x) = Rx$ for every $x\in E$, and $\Vert {\widehat{R}}\Vert =\Vert R\Vert \leqslant M^{(p)}(X)\,\Vert T\Vert$. Since $S\circ {\widehat{T}}$ and ${\widehat{R}}$ are lattice homomorphisms which agree on the set $\{\delta _x\,:\,x\in E\}$, it follows that $S\circ {\widehat{T}}={\widehat{R}}|_L$. Hence we have

$$\begin{aligned} \Vert {\widehat{T}} f\Vert _X= & {} x^*\bigl (|{\widehat{T}} f|\bigr ) =\bigl \Vert Q({\widehat{T}}f)\bigr \Vert _{L_1(\mu )} \leqslant \bigl \Vert S({\widehat{T}}f)\bigr \Vert _{L_p(hd\mu )}\\= & {} \Vert {\widehat{R}} f\Vert _{L_p(h\,d\mu )} \leqslant M^{(p)}(X)\,\Vert T\Vert \,\Vert f\Vert _p. \end{aligned}$$

$$\square $$

### Remark 6.2

We do not know of an explicit formula for the norm of the free Banach lattice with upper *p*-estimates, even if the constant is one. In fact, we do not know whether this space—or ${{\,\mathrm{\mathrm{FBL}}\,}}^{\mathcal {D}}[E]$ in general—can be realized as a lattice of functions on the ball of $E^*$. Formulating the latter question more rigorously, we ask: Is it true that lattice homomorphisms from ${{\,\mathrm{\mathrm{FBL}}\,}}^{\mathcal {D}}[E]$ to ${\mathbb {R}}$ separate the points of ${{\,\mathrm{\mathrm{FBL}}\,}}^{\mathcal {D}}[E]$?

## Nonlinear (*p*, *q*)-summing maps and applications

The purpose of this section is to explore the norm (1.1) further. It has an obvious similarity with the *p*-summing norm of a linear operator. A very substantial body of literature is devoted to the study of *p*-summing norms, their applications, and generalizations in the linear case. We refer to [11] for a comprehensive exposition of this theory. Our aim is to establish analogues of a few of these classical results in our setting.

We begin by introducing a more general version of the norm (1.1) involving two indices $1\leqslant p,q < \infty$ and investigating the fundamental properties of this new norm. For a Banach space *E* and a function $f\in H[E]$, define

7.1 $$\begin{aligned} \Vert f\Vert _{p,q} =\sup \biggl \{\Bigl (\sum _{k=1}^n \bigl |f(x_k^*)\bigr |^p\Bigr )^{\frac{1}{p}} \,:\,n\in {\mathbb {N}},\, x_1^*,\dots ,x_n^*\in E^*,\, \mu _{q}(x_1^*,\ldots ,x_n^*)\leqslant 1\biggr \}\nonumber \\ \end{aligned}$$

and

$$\begin{aligned} H_{p,q}[E] = \bigl \{ f\in H[E] \,:\,\Vert f\Vert _{p,q} < \infty \bigr \}. \end{aligned}$$

Denote by $\Vert \,\cdot \,\Vert _\infty$ the supremum norm on $B_{E^*}$, and let $H_\infty [E]$ be the sublattice of *H*[*E*] of all positively homogeneous functions which are bounded on $B_{E^*}$. Note that $\Vert f\Vert _\infty \leqslant \Vert f\Vert _{p,q}$ for every $1 \leqslant p,q < \infty$ and $f\in H[E]$, and consequently $H_{p,q} [E] \subseteq H_\infty [E]$. Note also that in the notation previously introduced, we have $\Vert f\Vert _p=\Vert f\Vert _{p,p}$ and $H_{p}[E]=H_{p,p}[E]$.

The following lemma is straightforward.

### Lemma 7.1

Given $1\leqslant p,q <\infty$ and a Banach space *E*, the space $\bigl (H_{p,q}[E],\Vert \,\cdot \,\Vert _{p,q}\bigr )$ equipped with the pointwise vector lattice operations is a *p*-convex Banach lattice with *p*-convexity constant one.

It is also easy to see that this space is of interest only for $p\geqslant q$.

### Lemma 7.2

Let $1\leqslant p<q<\infty$. Then $H_{p,q}[E]=\{0\}$ for every Banach space *E*.

### Proof

Let $p,q\in [1,\infty )$, and suppose that $H_{p,q}[E]$ contains a nonzero function *f*. Choose $x^* \in E^*$ such that $f(x^*)\ne 0$. Then, for every $n\in {\mathbb {N}}$, we have

$$\begin{aligned} n^{\frac{1}{p}}\bigl |f(x^*)\bigr | =\Bigl (\sum _{k=1}^n\bigl |f(x^*)\bigr |^p\Bigr )^\frac{1}{p} \leqslant \Vert f\Vert _{p,q}\cdot \mu _{q}(\,\underbrace{x^*,\ldots ,x^*}_n\,) =\Vert f\Vert _{p,q}\,n^{\frac{1}{q}}\,\Vert x^*\Vert , \end{aligned}$$

which implies that

$$\begin{aligned} n^{\frac{1}{p}-\frac{1}{q}} \leqslant \frac{\Vert x^*\Vert }{\bigl |f(x^*)\bigr |}\Vert f\Vert _{p,q}. \end{aligned}$$

Since the right-hand side is independent of *n*, we conclude that $\frac{1}{p}-\frac{1}{q}\leqslant 0$, that is, $p\geqslant q$. $\square$

Our next result provides the general comparison among these norms. The argument follows the same approach as in the Inclusion Lemma [11, 2.8].

### Proposition 7.3

Let $1 \leqslant q_j \leqslant p_j < \infty$ for $j=1,2$, and suppose that $p_1 \leqslant p_2,$ $q_1 \leqslant q_2,$ and $\frac{1}{q_1} - \frac{1}{p_1} \leqslant \frac{1}{q_2} - \frac{1}{p_2}$. Then

$$\begin{aligned} \Vert f\Vert _{p_2, q_2} \leqslant \Vert f\Vert _{p_1 , q_1} \end{aligned}$$

for every $f \in H[E]$. In particular, $H_{p_1,q_1} [E] \subseteq H_{p_2 , q_2}[E]$.

### Proof

We begin by observing that the result follows easily for $q_1 = q_2$, and if $p_1=p_2$, then the inequalities $q_1 \leqslant q_2$ and $\frac{1}{q_1} - \frac{1}{p_1} \leqslant \frac{1}{q_2} - \frac{1}{p_2}$ imply that $q_1 = q_2$. Thus, we may assume that $p_1< p_2$ and $q_1< q_2$, and then define

$$\begin{aligned} \frac{1}{p} = \frac{1}{p_1} - \frac{1}{p_2} , \quad \frac{1}{q} = \frac{1}{q_1} - \frac{1}{q_2}, \end{aligned}$$

which satisfy $1< p\leqslant q < \infty$ by the hypotheses.

Let $f\in H [E]$ and fix any $x_1^* , \dots , x_n^* \in E^*$ with $\mu _{q_2}(x_1^*,\ldots ,x_n^*)\leqslant 1$. For $1\leqslant k \leqslant n$, define $\lambda _k= \bigl |f(x_k^*)\bigr |^{p_2 / p}$. By the homogeneity of *f*, we have

7.2 $$\begin{aligned} \sum _{k=1}^n\bigl |f(x_k^*)\bigr |^{p_2} =\sum _{k=1}^n\bigl |f(\lambda _k x_k^*)\bigr |^{p_1} \leqslant \Vert f\Vert _{p_1,q_1}^{p_1}\,\mu _{q_1}(\lambda _1x_1^*,\ldots ,\lambda _nx_n^*)^{p_1}. \end{aligned}$$

Hölder’s inequality shows that

$$\begin{aligned} \Bigl (\sum _{k=1}^n\bigl |\lambda _k x_k^* (x)\bigr |^{q_1}\Bigr )^\frac{1}{q_1} \leqslant \Bigl (\sum _{k=1}^n \lambda _k^q\Bigr )^\frac{1}{q} \Bigl (\sum _{k=1}^n\bigl |x_k^* (x)\bigr |^{q_2}\Bigr )^\frac{1}{q_2} \leqslant \Bigl (\sum _{k=1}^n \lambda _k^q\Bigr )^\frac{1}{q} \leqslant \Bigl (\sum _{k=1}^n \lambda _k^p\Bigr )^\frac{1}{p} \end{aligned}$$

for every $x\in B_E$ because $\mu _{q_2}(x_1^*,\ldots ,x_n^*)\leqslant 1$ and $p\leqslant q$. Taking the supremum over $x\in B_E$ and using the definition of $\lambda _k$, we obtain

7.3 $$\begin{aligned} \mu _{q_1}(\lambda _1x_1^*,\ldots ,\lambda _nx_n^*) \leqslant \Bigl (\sum _{k=1}^n \bigl |f(x_k^*)\bigr |^{p_2}\Bigr )^{\frac{1}{p}}. \end{aligned}$$

We now substitute (7.3) into (7.2) and rearrange the inequality to conclude that

$$\begin{aligned} \Bigl (\sum _{k=1}^n \bigl |f(x_k^*)\bigr |^{p_2}\Bigr )^{1-\frac{p_1}{p}} \leqslant \Vert f\Vert _{p_1 , q_1}^{p_1}. \end{aligned}$$

This completes the proof because $1-\frac{p_1}{p}=\frac{p_1}{p_2}$. $\square$

### Proposition 7.4

Let *E* be a Banach space whose dual has finite cotype $r\geqslant 2,$ and suppose that $1 \leqslant q< p <\infty$ satisfy $\frac{1}{q} - \frac{1}{p} \geqslant 1-\frac{1}{r}$. Then $H_{p,q}[E] = H_\infty [E]$ with equivalence of norms.

### Proof

By [11, Corollary 11.17], every weakly summable sequence in $E^*$ is strongly *r*-summable, and there exists a constant $K > 0$ such that

$$\begin{aligned} \Bigl (\sum _{k=1}^n \Vert x_k^*\Vert ^{r}\Bigr )^\frac{1}{r} \leqslant K\mu _{1}(x_1^*,\ldots ,x_n^*) \end{aligned}$$

for every finite sequence $(x_k^*)_{k=1}^n$ in $E^*$. Hence, for $f\in H_\infty [E]$, we have

$$\begin{aligned} \Bigl (\sum _{k=1}^n\bigl |f(x_k^*)\bigr |^{r}\Bigr )^\frac{1}{r} \leqslant \Vert f\Vert _\infty \Bigl (\sum _{k=1}^n \Vert x_k^*\Vert ^{r}\Bigr )^\frac{1}{r} \leqslant K\, \Vert f\Vert _\infty \,\mu _{1}(x_1^*,\ldots ,x_n^*). \end{aligned}$$

Taking the supremum over all $n\in {\mathbb {N}}$ and $x_1^*,\dots ,x_n^*\in E^*$ with $\mu _{1}(x_1^*,\ldots ,x_n^*)\leqslant 1$, we conclude that $\Vert f\Vert _{r,1}\leqslant K\Vert f\Vert _\infty$.

Since $1 \leqslant q<p < \infty$ satisfy $\frac{1}{q} - \frac{1}{p}\geqslant 1 - \frac{1}{r}$, we can apply Proposition 7.3 with $p_2=p$, $q_2=q$, $p_1=r$, and $q_1=1$ to obtain $\Vert f\Vert _{p,q}\leqslant \Vert f\Vert _{r,1}\leqslant K\Vert f\Vert _\infty$, so that $f\in H_{p,q}[E]$ and the (*p*, *q*)- and supremum norms are equivalent. $\square$

As in the classical setting, the Dvoretzky–Rogers Theorem can be used to show that in general these norms are different:

### Proposition 7.5

Let *E* be an infinite-dimensional Banach space, and suppose that $1 \leqslant q \leqslant p <\infty$ satisfy $\frac{1}{q} - \frac{1}{p} < \frac{1}{2}$. Then $H_{p,q} [E] \subsetneq H_\infty [E]$.

### Proof

By the Dvoretzky–Rogers Theorem [11, Theorem 10.5], there exists a weakly *q*-summable sequence $(x_k^*)_{k\in {\mathbb {N}}}$ in $E^*$ which fails to be strongly *p*-summable. Now consider the function $f:E^*\rightarrow {\mathbb {R}}$ defined via $f(x^*)=\Vert x^*\Vert$. Clearly, $f\in H_\infty [E]$, and for every $n\in {\mathbb {N}}$, we have

$$\begin{aligned} \Bigl (\sum _{k=1}^n \Vert x_k^*\Vert ^p\Bigr )^\frac{1}{p} \leqslant \Vert f\Vert _{p,q}\, \mu _{q}(x_1^*,\ldots ,x_n^*). \end{aligned}$$

Letting $n\rightarrow \infty$, we see that $\Vert f\Vert _{p,q} = \infty$. Thus $f \not \in H_{p,q}[E]$. $\square$

Pietsch’s Domination Theorem (see, e.g., [11, Theorem 2.12]) is a cornerstone of the linear theory of *p*-summing operators with several important factorization results among its consequences. We conclude by providing analogues of [7, Propositions 2.12 and 2.13] for $1\leqslant p <\infty$.

Given a Banach space *E*, equip the unit ball $$B_{E^{**}}$$ of its bidual with the relative weak$$^*$$ topology, and denote the set of regular Borel probability measures on $$B_{E^{**}}$$ by $$\mathfrak P(B_{E^{**}})$$. This is a convex, weak$$^*$$ compact subset of the dual space of $$C(B_{E^{**}})$$. Each measure $$\mu \in \mathfrak P(B_{E^{**}})$$ induces a function $$f^p_\mu :E^*\rightarrow {\mathbb {R}}_+$$ via the definition

$$\begin{aligned} f_\mu ^p (x^*)=\biggl (\int _{B_{E^{**}}}\bigl |x^{**}(x^{*})\bigr |^p \, d\mu (x^{**})\biggr )^\frac{1}{p} \end{aligned}$$

for every $$x^*\in E^*$$. This provides a link between $$H_p[E]_+$$ and $${\mathfrak {P}}(B_{E^{**}})$$, as we now explain.

### Proposition 7.6

Let $$1\leqslant p <\infty $$ and $$\mu \in {\mathfrak {P}}(B_{E^{**}})$$. Then $$f_\mu ^p \in H_p[E]_+$$ with $$\Vert f_\mu ^p\Vert _p\leqslant 1$$.

### Proof

The function $f_\mu ^p$ is clearly positive and positively homogeneous. For $n\in {\mathbb {N}}$ and $x_1^*,\ldots , x_n^*\in E^*$, we have

$$\begin{aligned} \biggl (\sum _{k=1}^n\bigl |f_\mu ^p (x^*_k)\bigr |^p\biggr )^\frac{1}{p} =\biggl (\int _{B_{E^{**}}}\sum _{k=1}^n\bigl |x^{**}(x_k^*)\bigr |^p\, d\mu (x^{**})\biggr )^\frac{1}{p}\\ \leqslant \sup _{x^{**}\in B_{E^{**}}} \biggl (\sum _{k=1}^n\bigl |x^{**}(x_k^*)\bigr |^p\biggr )^\frac{1}{p} =\mu _{p}(x_1^*,\ldots ,x_n^*), \end{aligned}$$

where the last equality follows from the weak$$^*$$ density of $$B_E$$ in $$B_{E^{**}}$$. Hence $$\Vert f_\mu ^p\Vert _p\leqslant 1$$.

$$\square $$

### Proposition 7.7

Let $$1\leqslant p <\infty $$. For every $$f\in H_p[E]_+$$, there is a measure $$\mu \in {\mathfrak {P}}(B_{E^{**}})$$ such that

$$\begin{aligned} f(x^*)\leqslant \Vert f\Vert _p \, f_\mu ^p(x^*) \end{aligned}$$

for every $$x^*\in E^*$$.

### Proof

This proof is based on the proof of Pietsch’s Domination Theorem given in [11, 2.12]. For every nonempty finite subset *M* of $$E^{*}$$, define $$g_M :B_{E^{**}}\rightarrow {\mathbb {R}}$$ by

$$\begin{aligned} g_M(x^{**}) =\sum _{x^* \in M}\Bigl (f(x^*)^p -\Vert f\Vert _p^p \cdot \bigl |x^{**}(x^{*})\bigr |^p\Bigr ). \end{aligned}$$

Then $$g_M$$ is weak$$^*$$ continuous, and so the set *Q* of all such functions $$g_M$$ is contained in $$C(B_{E^{**}})$$. Given nonempty finite subsets $$M_1$$ and $$M_2$$ of $$E^{*}$$ and $$0<\lambda <1$$, the positive homogeneity of *f* implies that $$\lambda \cdot g_{M_1}+(1-\lambda )\cdot g_{M_2}=g_{M_3}$$, where

$$\begin{aligned} M_3= \bigl \{\lambda ^{1/p}x^* \,:\,x^* \in M_1 \bigr \} \cup \bigl \{(1-\lambda )^{1/p}x^* \,:\,x^* \in M_2\bigr \}. \end{aligned}$$

This shows that *Q* is a convex set.

The definition (1.1) of the norm $\Vert \,\cdot \,\Vert _p$ implies that *Q* is disjoint from the strictly positive cone

$$\begin{aligned} P=\Bigl \{h\in C(B_{E^{**}})\,:\,h(x^{**})>0\ \text {for every}\ x^{**}\in B_{E^{**}}\Bigr \}. \end{aligned}$$

Since *P* is open and convex, the geometric version of the Hahn–Banach Theorem guarantees the existence of a functional $$\mu \in C(B_{E^{**}})^{*}$$ and a constant $$c\in {\mathbb {R}}$$ such that $$\mu (g)\leqslant c<\mu (h)$$ for every $$g\in Q$$ and $$h\in P$$.

Choosing $$M=\{0\}\subseteq E^*$$, we have $$g_M=0$$. Therefore $$0\in Q$$, and so $$c\geqslant 0$$. On the other hand, as every strictly positive constant function belongs to *P*, we must have $$c\leqslant 0$$. It follows that $$c=0$$, which implies that $$\mu (h)\geqslant 0$$ for every $$h\in C(B_{E^{**}})_{+}$$. In other words, $$\mu $$ is a positive regular Borel measure such that

$$\begin{aligned} \int _{B_{E^{**}}} g\,d\mu \leqslant 0<\int _{B_{E^{**}}} h\, d\mu \end{aligned}$$

for every $$g\in Q$$ and $$h\in P$$. This inequality is unaffected by scaling of $$\mu $$, so we may assume that $$\mu \in {\mathfrak {P}}(B_{E^{**}})$$. For every $$x^* \in E^*$$, the function $$g_{\{x^*\}}$$ belongs to *Q*, and therefore

$$\begin{aligned} 0\geqslant \int _{B_{E^{**}}}\Bigl (f(x^*)^p -\Vert f\Vert _p^p \cdot \bigl |x^{**}(x^{*})\bigr |^p\Bigr ) d\mu (x^{**}) =f(x^*)^p - \Vert f\Vert _p^p\, f_\mu ^p(x^*)^p \end{aligned}$$

because $\mu$ is a probability measure. $\square$

We can summarize the conclusions of Propositions 7.6 and 7.7 as follows.

### Corollary 7.8

Let $$1\leqslant p <\infty $$ and $$f\in H[E]_+$$. Then $$f\in H_p[E]_+$$ if and only if, for some constant $$C>0,$$ there is a measure $$\mu \in {\mathfrak {P}}(B_{E^{**}})$$ such that

$$\begin{aligned} f(x^*)\leqslant C\cdot f_\mu ^p(x^*) \end{aligned}$$

for every $x^* \in E^*$. Furthermore, when $f\in H_p[E]$, its norm $\Vert f\Vert _p$ can be computed as the infimum of all constants *C* for which such a measure $\mu$ exists.
